# Understanding the Chronology and Occupation Dynamics of Oversized Pit Houses in the Southern Brazilian Highlands

**DOI:** 10.1371/journal.pone.0158127

**Published:** 2016-07-06

**Authors:** Jonas Gregorio de Souza, Mark Robinson, Rafael Corteletti, Macarena Lucia Cárdenas, Sidnei Wolf, José Iriarte, Francis Mayle, Paulo DeBlasis

**Affiliations:** 1 Department of Archaeology, University of Exeter, Exeter, United Kingdom; 2 Museu de Arqueologia e Etnologia, Universidade de São Paulo (MAE-USP), São Paulo, Brazil; 3 Department of Geography and Environmental Science, University of Reading, Reading, United Kingdom; 4 Centro Universitário Univates, Lajeado, Brazil; Seoul National University College of Medicine, REPUBLIC OF KOREA

## Abstract

A long held view about the occupation of southern proto-Jê pit house villages of the southern Brazilian highlands is that these sites represent cycles of long-term abandonment and reoccupation. However, this assumption is based on an insufficient number of radiocarbon dates for individual pit houses. To address this problem, we conducted a programme of comprehensive AMS radiocarbon dating and Bayesian modelling at the deeply stratified oversized pit House 1, Baggio I site (Cal. A.D. 1395–1650), Campo Belo do Sul, Santa Catarina state, Brazil. The stratigraphy of House 1 revealed an unparalleled sequence of twelve well preserved floors evidencing a major change in occupation dynamics including five completely burnt collapsed roofs. The results of the radiocarbon dating allowed us to understand for the first time the occupation dynamics of an oversized pit house in the southern Brazilian highlands. The Bayesian model demonstrates that House 1 was occupied for over two centuries with no evidence of major periods of abandonment, calling into question previous models of long-term abandonment. In addition, the House 1 sequence allowed us to tie transformations in ceramic style and lithic technology to an absolute chronology. Finally, we can provide new evidence that the emergence of oversized domestic structures is a relatively recent phenomenon among the southern proto-Jê. As monumental pit houses start to be built, small pit houses continue to be inhabited, evidencing emerging disparities in domestic architecture after AD 1000. Our research shows the importance of programmes of intensive dating of individual structures to understand occupation dynamics and site permanence, and challenges long held assumptions that the southern Brazilian highlands were home to marginal cultures in the context of lowland South America.

## Introduction

Archaeologists have for a long time debated the degree of permanence in the pit house villages of the southern Brazilian highlands [1–3]. A frequent assumption is that these sites are the result of cycles of short-term occupations separated by long periods of abandonment [1, 2]. Such portrayals, emphasising high mobility and low population levels, adhere to a long-held view that this area was marginal in the context of lowland South America [4] and fail to adequately evaluate the degree of social complexity among these societies in the pre-Columbian past. A proper understanding of household organization and occupation dynamics in pit house sites has been hampered until now by the absence of an adequate number of radiocarbon dates. Most of the discussions about site permanence in the southern Brazilian highlands were based on single dates for selected strata of isolated pit house structures in different sites [1, 2, 5].

To address these shortcomings, we designed a programme of comprehensive dating of an oversized, deeply stratified pit house in the southern Brazilian highlands: House 1, Baggio I site. The excavation of the site uncovered an unparalleled sequence of twelve floors among which we documented noticeable changes in the tempo and practices of refurbishment events, as well as in lithic technology and ceramic style. We obtained a corpus of eleven AMS radiocarbon dates, which were modelled through Bayesian statistics, allowing us for the first time to time those changes within a well-defined chronology for a single structure.

### The southern proto-Jê groups

The pit houses of the southern Brazilian highlands, together with other forms of earthen architecture, including circular enclosures, linear walls, and mounds, are distinctive of the Taquara-Itararé Tradition. This archaeological tradition was broadly defined based on a simple association between diagnostic small ceramics and these earthworks [6–8]. We now recognise a large diversity within the Taquara-Itararé Tradition in ceramics, settlement patterns and site types. The latter include not only earthworks, but also surface litho-ceramic sites, rock shelters, rock art sites, coastal shell middens, and reoccupations of Mid Holocene shell mounds [3, 6–10]. The Taquara-Itararé Tradition started to spread throughout southern Brazil around Cal. 380 B.C. and endured until the 17^th^ century, presenting continuity with the historical indigenous groups of the region. These modern groups, the Kaingang and Xokleng, belong to the southern branch of the Jê linguistic family [7]. In order to emphasise the continuity from the archaeological record to the present, we follow Iriarte *et al*. [11] in referring to the prehistoric groups as the *southern proto-Jê*, in which ‘proto’ encompasses all ancestors of the modern groups, including the extinct Kimdá and Ingáin [12].

Although the southern proto-Jê groups occupied a range of environments in southern Brazil and adjacencies, earthworks are primarily found in the basaltic highlands, predominantly above 600 m of elevation, coinciding with the distribution of the *Araucaria angustifolia* (Paraná pine) forests that were of prime economic importance for the Jê groups described in historical accounts (17^th^ century to the present).

The class of earthworks that received most attention in recent times are the ceremonial mound and enclosure complexes. These are circular or, rarely, rectangular earthworks with diameters ranging between 15 m and 180 m, and may or may not include central mounds [11, 13–15]. Mound and enclosure complexes are carefully placed in the landscape, usually on hill tops, in order to command a wide viewshed [11, 16]. Central mounds are funerary structures with secondary cremated burials and, more rarely, remains of funeral pyres [15, 17, 18]. In more intensely surveyed areas, mound and enclosure complexes have been found to be part of highly structured landscapes, where they always occur in the vicinity of pit house sites [11, 16].

Pit houses first appear in the highlands around Cal. A.D. 415 [6, 19]. There were until now few dates posterior to the 16^th^ century A.D., which suggested a gap between that form of domestic architecture and the earliest European accounts of the southern Jê groups in the 17^th^ century [20]. These structures appear mostly isolated or in small groups, with some exceptional clusters of up to 107 pit houses [1, 6]. The structures are circular or elliptical, and normally do not exceed 5 m in diameter [6]. However, there are oversized houses that can attain more than 25 m diameter and up to 7 m depth, frequently in the vicinity of mounds up to 2 m high [5, 21]. Oversized pit houses are commonly found isolated from other houses, but sometimes form part of larger settlements, in which case they are often found in a central position, surrounded by smaller pit houses [19, 21, 22].

### Previous research on oversized pit houses

The function of oversized pit houses has been widely debated, but few of these structures have actually been excavated and dated. In the municipality of Vacaria, Rio Grande do Sul state, Schmitz *et al*. [2] excavated oversized houses at two sites, RS-A-27 and RS-A-29. At site RS-A-27, the excavation of House 3 (14 m diameter) revealed no clear activity areas, few artefacts in proportion to the large dimensions of the structure, and a tendency for an increase in the quantity of ceramics over time. The fact that few artefacts were found suggests that the house was kept clear of debris and ceramic refuse was not incorporated into construction fill. House 2 (14.5 m diameter), at site RS-A-29, also had few artefacts—in fact, it was the cleanest house in the site—but the recovered material did include lithics of good quality raw materials [2].

At Bom Jesus, close to the contexts excavated by Schmitz *et al*., Copé [21] excavated an oversized pit house (House A, 18 m diameter) at site RS-AN-03. A semicircle of five hearths associated with ceramics and lithics was found around the central post holes of the house. This arrangement of hearths in semicircle is interpreted by Copé [21] as the material remains of recurrent gatherings around a central space, suggesting that House A could have been either the dwelling of a high-status individual who hosted meetings, or purely a communal facility.

The oversized pit houses of São José do Cerrito are the closest geographically to our study area. They were originally surveyed by Reis [23], who was interested in the possible communal function of oversized pit houses. She noticed that such structures were rare and tended to occur isolated from other pit houses and far from other sites. A review of the radiocarbon dates then available led her to propose that the larger pit houses were earlier than smaller ones, representing an extended family residential pattern—later replaced by settlements with many small dwellings for nuclear families. Reis [23] also excavated an oversized pit house (SC-CL-52, 20 m diameter), noticing that it contained very few artefacts, but without providing a description of the stratigraphy or distribution of the finds.

More recently, the region of São José do Cerrito was revisited by Schmitz *et al*. [5], who obtained dates for SC-CL-52 and other sites. The oversized pit house of SC-CL-52 was dated 860 ± 30 ^14^C BP (Cal. A.D. 2σ 1175–1275), whereas smaller pit houses in the vicinity (sites SC-CL-43 and SC-CL-51) were dated between 370 ± 40 (Cal. A.D. 2σ 1460–1640) and 320 ± 30 ^14^C BP (Cal. A.D. 2σ 1500–1665). These dates led Schmitz *et al*. [5] to endorse the hypothesis that large pit houses were earlier than small ones. Reaffirming the findings of Reis [23], they also found very few artefacts in the interior of the oversized pit house SC-CL-52, which the authors interpret as disproportional in comparison to the energy invested in its construction. It should be kept in mind, however, that this could be the result of regular cleaning. In reference to labour, Schmitz *et al*. [5] conclude that the labour necessary for the construction of site SC-CL-52 suggests the occupation by an extended family or even larger group.

### Pit house occupation dynamics

There were, until now, few dates to inform debates about the degree of continuous occupation in pit houses. As Saldanha [16] and Iriarte *et al*. [11] point out, the careful planning evident in some site layouts, coupled with earthworks such as outer terraces and trackways, point toward the contemporaneous occupation of multiple houses through a long time period, but the absence of dates hampers a definitive evaluation of that hypothesis. Copé [21] argues that the stratigraphies of two pit houses at site RS-AN-03 show no periods of abandonment. The dates obtained for the base and top of the occupation layers of House C are 1070 ± 70 ^14^C BP (Cal. A.D. 2σ 880–1180) and 550 ± 40 ^14^C BP (Cal. A.D. 2σ 1325–1455). Copé [21] argues that they point to over five centuries of history at the site, but a larger number of dates, ideally from all identified strata, would be preferable to reinforce that hypothesis. On the other hand, Schmitz *et al*. argue in several works [1, 2, 5] that long cycles of abandonment and reoccupation existed, but the radiocarbon dates presented are insufficient to support that conclusion, with few dates obtained within selected pit houses from different sites. For example, at site RS-A-29, with 40 pit houses, single strata from only four different houses were dated [2]. The dates, varying between 710 ± 60 ^14^C BP (Cal. A.D. 2σ 1230–1405) and 370 ± 50 ^14^C BP (Cal. A.D. 2σ 1455–1645), were interpreted by Schmitz *et al*. [2] as representing a palimpsest of periodic abandonment and return to the site, with new houses built at each stage. However, because only a single date exists for each of a small number of houses, we cannot preclude the possibility that old houses were continuously occupied as new ones were built in their vicinity, as expected in a normal process of sedentary village growth.

Our work in House 1 of the Baggio I site in the context of the AHRC-FAPESP project “Jê Landscapes of southern Brazil: Ecology, History and Power in a Transitional Landscape during the Late Holocene” is the first to fill the gap in pit house chronology. House 1, an oversized pit house with a 16 m diameter and 1.6 m depth before excavation, provides the first robust pit house chronology of the southern Brazilian highlands, with AMS radiocarbon dates for nine of twelve successive floors.

## Materials and Methods

All necessary permits were obtained for the described study, which complied with all relevant regulations. The permit to excavate archaeological sites in the municipality of Campo Belo do Sul was granted by IPHAN (Brazilian National Institute of Artistic and Historic Heritage). The excavations in the property of Mr. Valmor Baggio have been conducted with the kind permission of the land owner. No other permits were required. All artefacts were collected, analysed and deposited at UNISUL (Universidade do Sul de Santa Catarina), Tubarão, Santa Catarina state, Brazil, where they were inventoried and are publicly accessible. The finds are identified by site (Baggio I), structure (House 1), unit (Area A/B), level and individual collection number.

### The Baggio I site

**Fig 1:**
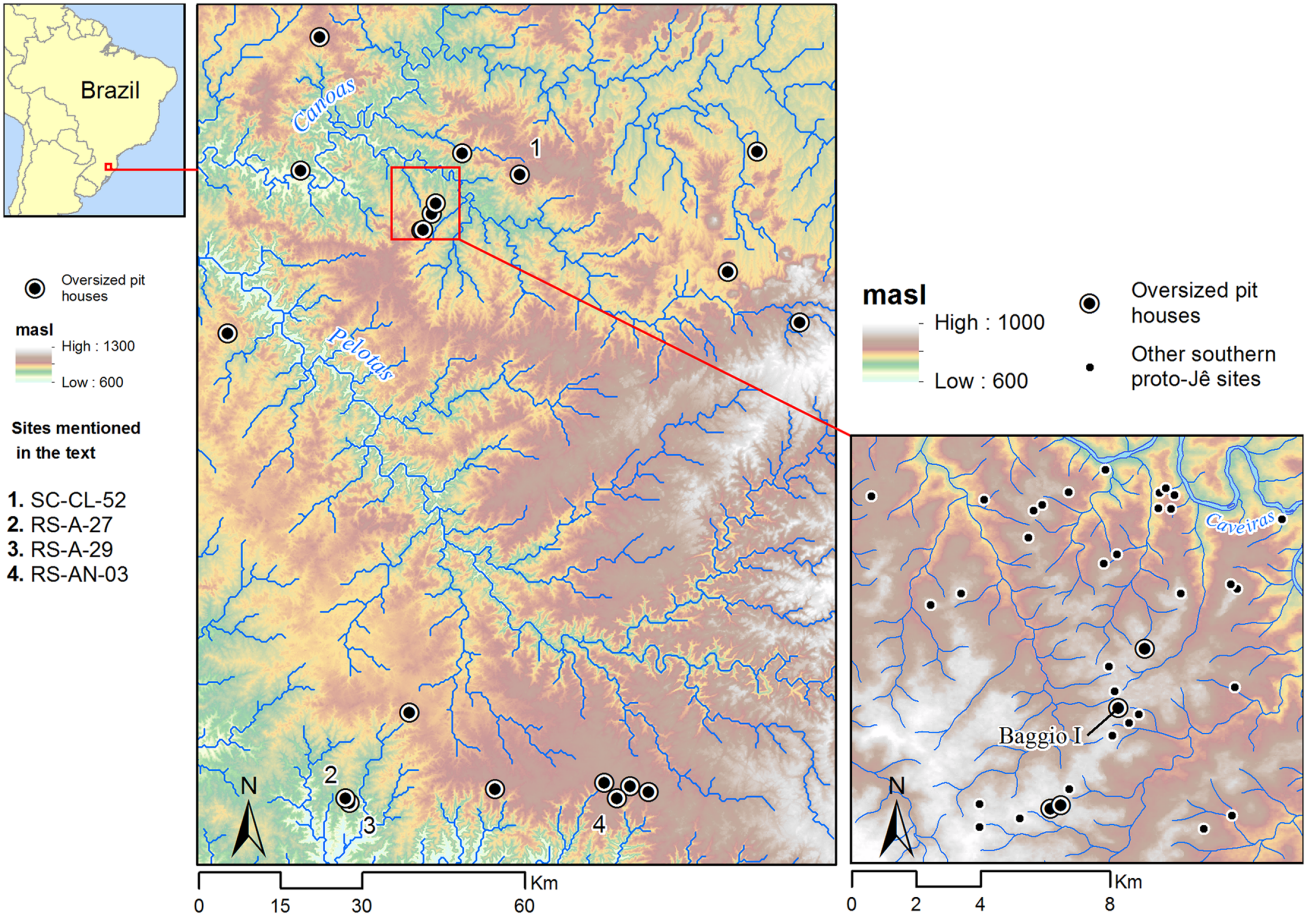
Map of the study area. Oversized pit houses in the southern Brazilian highlands close to the study area are also shown. In detail, the location of Baggio I is indicated together with other sites surveyed in Campo Belo do Sul.

The Baggio I site was first identified during a survey conducted to the south of the Caveiras river, in the municipality of Campo Belo do Sul, Santa Catarina state. The site is located in the Canoas-Pelotas basin, a region where one can find the highest density of southern proto-Jê sites, a long history of occupation (with some of the earliest and latest dates), and great variability in both domestic and ceremonial earthen architecture [1, 5, 13, 23]. This region also concentrates a large number of oversized pit houses, including Baggio I and others recorded during our survey (Fig 1).

**Fig 2:**
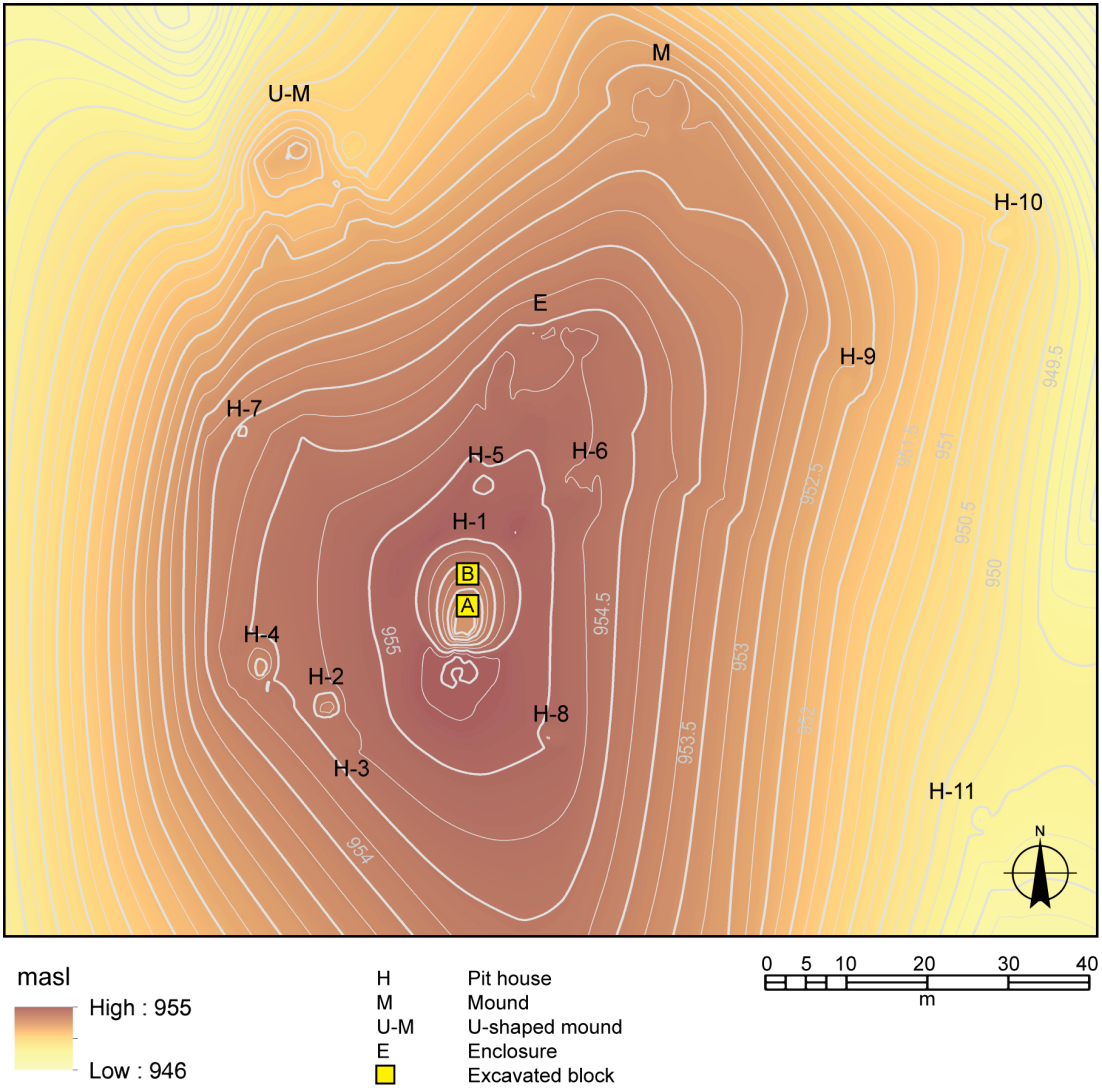
Digital elevation model of the inner precinct of Baggio I.

The Baggio I site (Fig 2) can be divided into an inner precinct with formal architectural arrangement and a peripheral area with dispersed, less formal patterning in architecture. The inner precinct occupies an area of 2 ha on a hilltop, and exhibits the pit house with the largest diameter (16 m) and depth (1.6 m), henceforth called House 1, surrounded by seven smaller pit houses, between 2 m and 5 m diameter. A platform is located 60 m northwest, downhill from House 1. This platform is flanked by two low parallel arms, giving it a U shape facing House 1. This is a novel form of mound architecture never recorded before in the southern Brazilian highlands, and all the more interesting since its orientation seems to reference House 1. Adjacent to House 1, to the east, is located a small circular enclosure (14 m diameter). A further eight pit houses (2.5 m to 7 m diameter) occur in the lower slopes of the hill to the southeast.

We targeted different structures of the site during the first field season. However, because the focus of this paper is to discuss house permanence and occupation dynamics, we will present the results of the excavation of House 1 because of its clearly defined if complex stratigraphy, number of radiocarbon dates obtained, and rich artefact assemblage. Two 2 x 2 m units (named Area A and Area B) were excavated near the centre of House 1, separated by a 1 m unexcavated bulk (Fig 2). The excavation revealed a sequence of twelve well-defined floors.

### Stratigraphy of House 1

**Fig 3:**
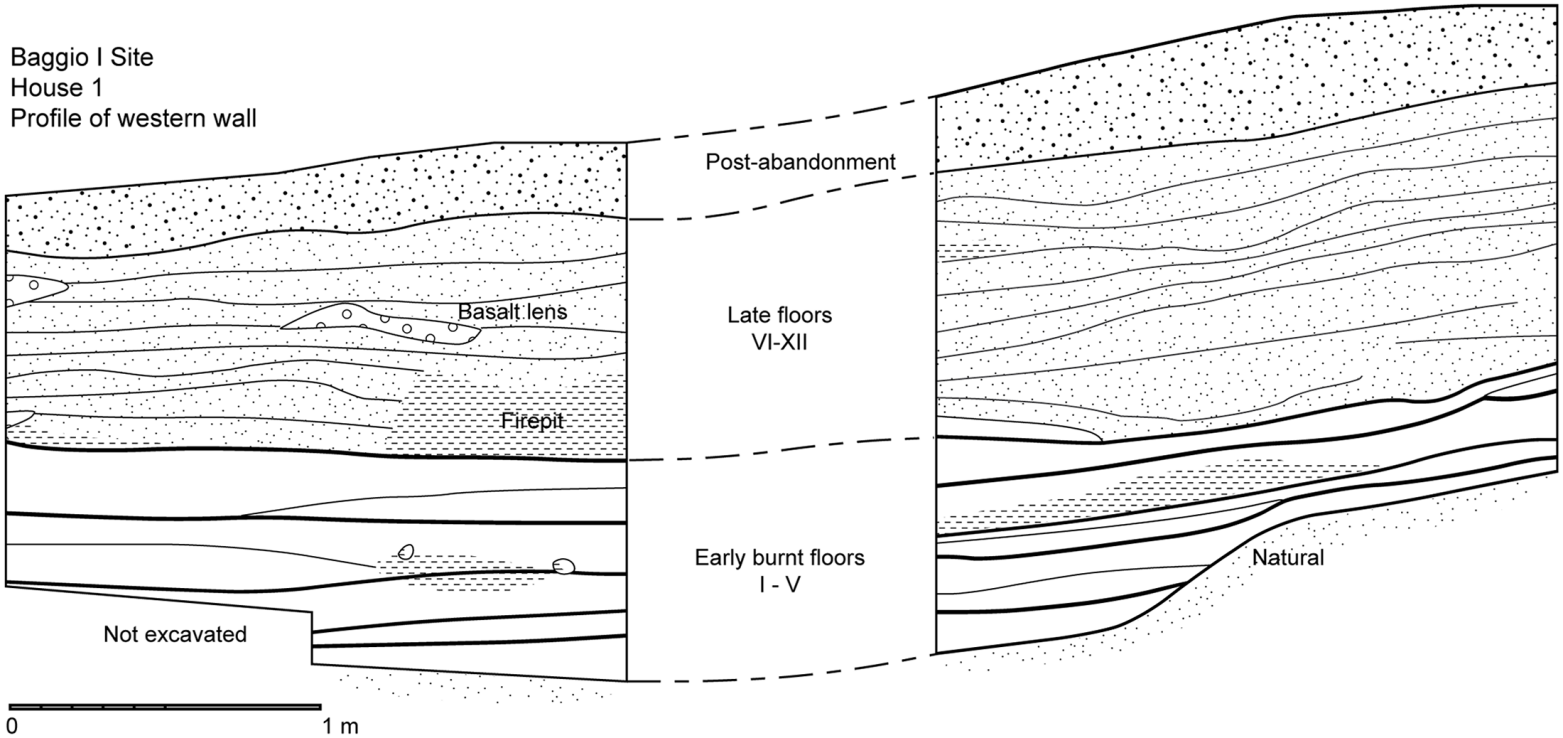
Profile of western wall of House 1, Areas A and B.

**Fig 4:**
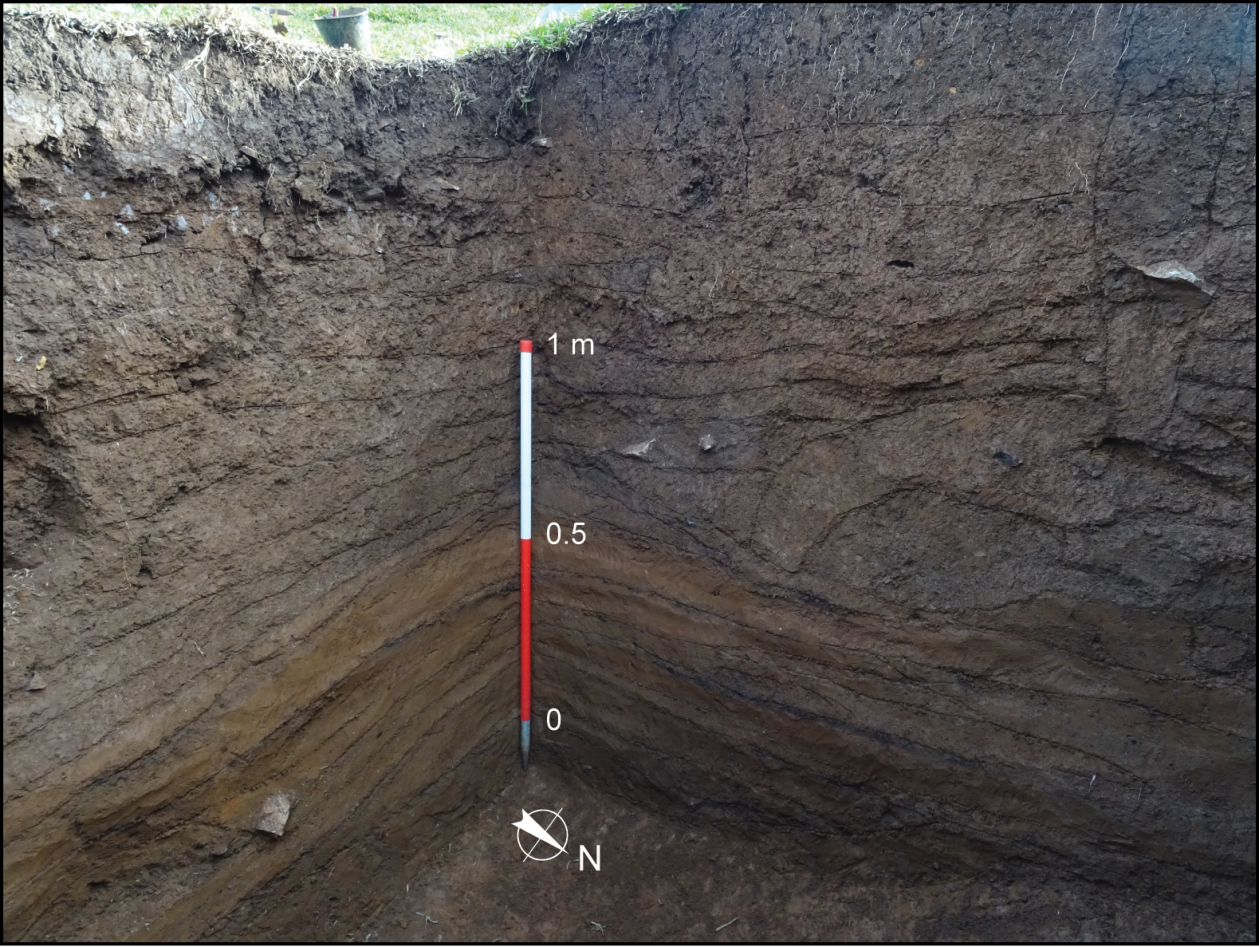
Photograph of western and southern walls of Area B, House 1.

The stratigraphic sequence of House 1 can be divided, from bottom to top, into (i) an early sequence of five heavily burnt floors, followed by (ii) a late sequence of seven floors, mostly clean of debris, and finally (iii) the post-abandonment layer (Figs 3 and 4).

**Fig 5:**
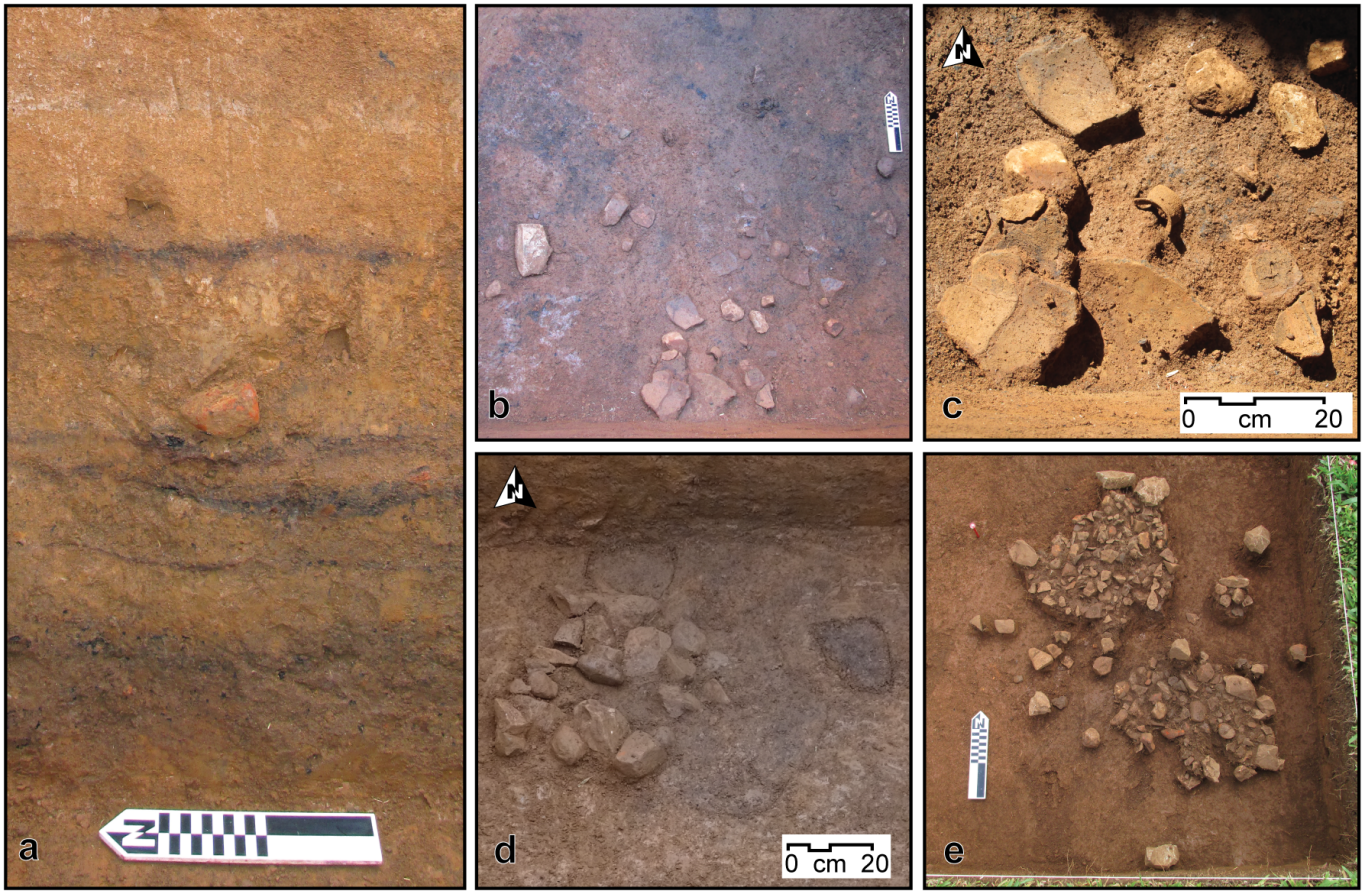
Detail of the stratigraphy, features, and caches excavated in House 1. a) Detail of the sequence of burnt floors in the profile of Area A. b) Floor 3, Area B, with ceramic cache. c) Detail of the ceramic cache of Floor 3, Area B. d) Stone cluster interpreted as a fire pit on Floor 6, Area B. e) Stone clusters interpreted as fire pits on Floor 12, Area B.

The five earliest floors consisted of thin surfaces littered with charcoal. On top of the charcoal lay large ceramic sherds, as well as lithics and stone clusters (Fig 5a–5c). The charcoal that covered these floors consisted of charred intertwined fibres, interpreted as remnants of thatch from the roof of the structure. The sherds found on top of the burnt surface were large, and sometimes articulated and belonging to the same vessel. They lay directly on top of the burnt surfaces, and therefore cannot represent *de facto* or primary refuse, but must have been deposited after the roof was set on fire and collapsed. This conclusion is reinforced by the fact that the artefacts were not burnt throughout, but only on the down facing surfaces that adhered to the burnt surface—i.e., they must have been added after the burning of the structure.

The burnt surfaces did not follow the modern inclination of the terrain, but sloped considerably towards the east, suggesting that the original architecture of the house differed from the present-day topography of the structure. The thin charcoal layers were separated by a matrix of hard packed orange clay (Fig 5a). Of particular interest was Floor 3, which contained a cache of ceramics, including a small decorated cup (Fig 5b and 5c), and a concentration of stones, burnt logs, columnar basalt and large ceramics. Charred bark was found amidst the ceramic cache, and a variety of carbonised botanical material was identified on this floor, including *Araucaria angustifolia* nodes and charred palm fibres. The deepest floor (Floor 1) lay on the transition to the natural clay, corresponding to the original cut of the pit house, and was lined with burnt cobbles associated with the charred fibres from the roof.

The last burnt floor marked the transition to a sequence of seven floors where the practice of collapsing and setting fire to the roof was no longer observed. These later floors were recognised solely by changes in colour and texture: each consisted of a continuous layer of hard packed clay that followed the slope of the terrain. These surfaces were intercalated with loose clay sediments without inclusions, representing infilling events between the occupations. On top of the compact surfaces lay charcoal, artefacts and features (burnt patches, lenses of degraded basalt and stone clusters) and they were sometimes cut by features such as post holes (Fig 5d). With the exception of Floor 12, all floors contained remarkably few artefacts. Floor 12, the last floor before abandonment, had two small stone clusters interpreted as stone-lined fire pits, associated with charcoal and concentrations of artefacts within them and in their close proximity (Fig 5e), including a stone scraper, many lithic flakes and small ceramic sherds. The artefacts represent primary debris left to accumulate around the cooking facilities and trampled into the floor in a moment close to the terminal abandonment of House 1, when the regular cleaning evident in the previous floors was no longer practiced.

Finally, on top of Floor 12, the post-abandonment stratum consisted of the humic layer and top soil, with silty clay sediments, recent charcoal from modern land clearance, small roots and loose lithics and ceramic sherds whose dispersal suggests they were slope-washed into the structure after its terminal abandonment.

It was clear from the stratigraphy that there were no major gaps in which House 1 would have lain abandoned for prolonged periods of time. The homogeneous sediments between the occupation floors were mostly clear of debris, resulting from rapid intentional fill episodes from single clay sources to prepare new habitation surfaces. This contrasts with the post-abandonment levels which contained appreciable quantities of loose lithics and ceramics amid the slowly accumulated and turbated natural soil formation and humic sediments. The unbroken floors and absence of soil development between them, coupled with the lack of materials that would have been washed from outside of the structure, is a strong indication that the house was not left vacant for long periods of time until its abandonment.

### Bayesian modelling

Bayesian statistical modelling consists in the incorporation of prior information, generally the known stratigraphic order of a sample of radiocarbon dates, in the estimation of the probable date range [24–26]. For example, if there is overlap between the calibrated ranges of two dates, but one is known with certainty to come from an earlier context than the other, those ranges can be narrowed with a greater precision. Thus, the combination of stratigraphy and calibrated radiocarbon dates provides a result that is more reliable than each of those lines of evidence considered in isolation [27]. In cases where a large number of radiocarbon dates are available and the knowledge about their stratigraphic relationship is secure, Bayesian modelling permits the construction of high-resolution chronologies, as demonstrated by a number of successful applications worldwide, where fine-grained chronologies sometimes attain the precision of a human generation [28–33].

In the case of the southern proto-Jê pit houses, it is not uncommon to find structures with over 1 m of stratified cultural deposits, their lower and upper strata separated by as much as five centuries [2, 19, 21]. Such stratified house structures, with multiple phases of construction, represent a fertile opportunity for the application of Bayesian modelling, allowing the assessment of household occupation dynamics within a fine-grained absolute chronology [33, 34]. Coupled with an understanding of a site’s macro- and micro-strata, this permits us to shed light on the social tempo and the collective rhythms expressed in recapping and refurbishing events [35]. However, this line of inquiry has so far never been pursued in the archaeology of southern Brazil, due to the fact that it demands a robust corpus of absolute dates.

**Fig 6:**
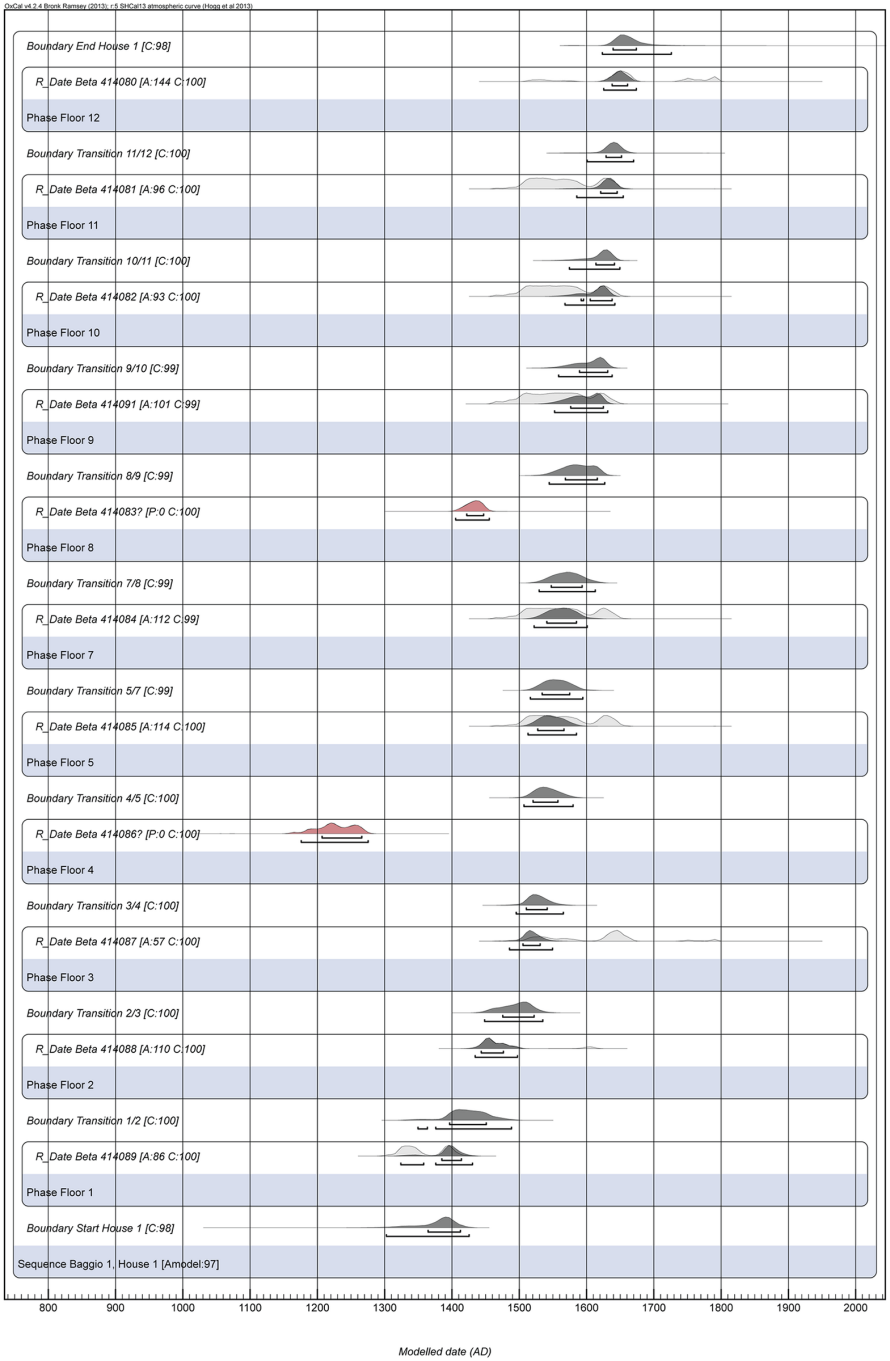
Bayesian model of the dates from House 1. The unmodelled probability distributions are shown as light grey areas, and the results of the Bayesian model appear as dark grey areas. Bars under each distribution represent 1σ and 2σ. Outliers, shown in red, have been calibrated but not included in the model. C = convergence, A = agreement index of each date, Amodel = overall agreement index of the model.

We performed Bayesian modelling of eleven radiocarbon dates (Table 1, Fig 6) from a sequence of twelve floors in House 1 using OxCal v4.2.4 [26, 36] and the southern hemisphere calibration curve (SHCal13) [37]. Charcoal from secure contexts in each floor was collected and sent to Beta Analytic for radiocarbon dating. We only dated charcoal that was directly on top of the floors or came from features that we interpreted as fire pits, hearths and collapsed roofs. All samples consist in charred material, received the standard Acid/Alkali/Acid pretreatment, and were dated by AMS. Eleven dates, from all but one floor, were thus obtained and initially entered into the Bayesian model.

**Table 1:** Modelled dates from House 1. Dates marked with * are outliers and were not included in the final run of the model. All dates are rounded to the nearest 5 years.

Stratum	Context	Laboratory number	Conventional Radiocarbon Age BP	δ13C %o	Cal A.D. (1σ)	Cal A.D. (2σ)	Median	Agreement	Convergence
Upper boundary					1635–1675	1620–1730	1660		96.4
Floor 12	Charcoal from fire pit	Beta 414080	280 ± 30	-25.2	1635–1665	1625–1675	1650	143.2	99
Floor 11	Charcoal on floor	Beta 414081	340 ± 30	-23.5	1620–1645	1585–1655	1630	96.6	99.6
Floor 10	Charcoal on floor	Beta 414082	350 ± 30	-28.9	1585–1640	1560–1645	1620	93.6	99.5
Floor 9	Charcoal on floor	Beta 414091	360 ± 30	-27.0	1575–1625	1550–1635	1595	101.2	99.2
Floor 8*	Charcoal on floor	Beta 414083	520 ± 30	-22.6	1420–1450	1405–1455	1435		99.1
Floor 7	Charcoal on floor	Beta 414084	350 ± 30	-24.7	1540–1585	1520–1605	1565	111.5	99.2
Floor 5	Charcoal from burnt roof	Beta 414085	340 ± 30	-27.4	1525–1570	1510–1585	1545	114.4	99.3
Floor 4*	Charcoal from burnt roof	Beta 414086	860 ± 30	-23.8	1205–1265	1175–1275	1225		98.9
Floor 3	Charcoal from burnt roof	Beta 414087	300 ± 30	-26.3	1505–1535	1485–1550	1520	57.4	99.7
Floor 2	Charcoal from burnt roof	Beta 414088	460 ± 30	-24.1	1440–1480	1430–1500	1460	110.1	99.8
Floor 1	Charcoal from burnt roof	Beta 414089	630 ± 30	-24.8	1380–1420	1315–1430	1395	87	99.1
Lower boundary					1360–1415	1300–1425	1385		95.3

We chose to obtain a vertical sequence of dates, rather than multiple dates for a single, or a few layers, due to the lack of a well-dated stratigraphic sequence from a single structure in the region. We recognise the limitations of using a single date per floor, since the results will not provide the duration of each episode of occupation; however, within the well-defined stratigraphic sequence they do allow us to estimate the approximate intervals between episodes and assess the chronology of occupation dynamics (for examples of previous research using similar datasets with good results, see [33, 38, 39]). We are confident that each dated sample represents a different stage of occupation of House 1. Each sample was collected from a discrete stratigraphic layer, separated by construction fill material. The homogenous material of each fill event demonstrates the rapid construction of each remodelling, utilising a single, culturally sterile clay source. The distinct boundary between the intact floors and construction fill events evidences the continuous occupation of the structure without periods of vacancy, which would result in bioturbation, soil formation, or other deposition, such as slope wash.

Each radiocarbon date was included in the model as a phase in a sequence of contiguous phases delimited by the simple boundary command based on the lack of gaps or overlaps between the occupation floors [26] (S1 Appendix). This is the model that most adequately reflects the stratigraphy of House 1. We also used the simple boundary command in OxCal for the start and end limits of the sequence of House 1, but had no material culture (such as a detailed ceramic style chronology for the area) or historical evidence to assume specific dates as priors. We know that the earliest radiocarbon date corresponds to the first burnt roof and thus provides a *terminus ad quem* for the original cut of the pit house; similarly, the latest sample comes from one of the fire pits on the most recent floor, but might not correspond to the cessation of use of that surface. Therefore, we relied on the results of the model for the start and end boundaries.

## Results and Discussion

### Chronology of House 1

OxCal facilitates the evaluation of the results by presenting an agreement index (A) that measures how well each date fits the model, as well as the likelihood of the model as a whole (A_model_). The initial run identified two dates (Beta 414083 and Beta 414086) as outliers based on the recommended agreement index threshold of 60%, and the model would not run with the inclusion of those dates [26] (Table 1, Fig 6). These two outliers were then excluded from the subsequent run of the model. One of the remaining dates (Beta 414087) had an agreement of 57.4%, only marginally inferior to the threshold. This date comes from a secure burnt roof context and does not affect the overall agreement of the model; based on those criteria, it should not be rejected [40].

The presence of outliers may be due to redeposition or old wood effect, a problem to be kept in mind when dating wood charcoal. However, most charcoal is expected to be only slightly earlier that its context of deposition, with a few older dates from old wood/redeposited charcoal [40]. Whereas that may be the case of the two identified outliers, the remaining dates provided a sequence that is not only coherent but also historically plausible (ending in the mid 17^th^ century).

The final model included nine radiocarbon dates and had an overall agreement index of 96.7% (Table 1). Bayesian modelling considerably narrowed the error ranges of the radiocarbon dates from an average of ± 109 years to ± 42 years at a 2σ confidence interval (Table 1, Fig 6).

In addition to providing the full probability distribution of calibrated radiocarbon dates, we also report point estimates in Table 1. Parameters such as the intercept, the mean and the median are commonly used for that purpose, but one must keep in mind that they are problematic for multimodal distributions, as they may fall in low probability regions [41, 42]. In Table 1, besides the 1σ and 2σ ranges, we report the median as a point estimate of the calendar age of each floor. All dates are rounded to the nearest 5 years. Whenever we discuss specific dates of House 1 along the text, we will be referring to the 2σ range.

We can confidently frame the occupation of House 1 between Cal. A.D. 1395 and 1650. Can we identify any significant hiatus along the history of use of the structure? Because we obtained single dates for each floor, we can only estimate the intervals between events, but not the duration of events. Nevertheless, those intervals are short– 60–65 years separating the earliest three floors, and 15–30 years between the subsequent floors (using the median as a base). Stratigraphic information precludes the possibility that those intervals correspond to periods of abandonment, due to the lack of soil formation, slope-washed materials or bioturbation between the habitation surfaces. These were rather separated by fill materials, especially evident in the first five episodes of occupation, which were intercalated by thick intentional deposits of hard packed sterile clay. Therefore, we interpret the intervals between the dates as the approximate time elapsed from one resurfacing episode to the next. The complete chronology of House 1 certainly shows no major periods of abandonment, contradicting earlier models that envisaged southern proto-Jê groups as highly mobile and adopting strategies of century-long cycles of intermittent occupations throughout their territories [2, 43].

#### Consequences for regional chronology

Previous research in the southern Brazilian highlands concluded, in the absence of absolute dates, that oversized pit houses represented multifamily dwellings from an early period in the history of the southern Jê groups, and that these were eventually replaced by small pit houses of nuclear families [23]. Radiocarbon and thermoluminescence dates now available confirm that, in many cases, large pit houses can be earlier than small ones within specific regions [5] or sites [2]. However, this is not true for all cases: within the same settlement, oversized pit houses can represent later additions [21]. Tables 2 and 3 summarise all available dates for oversized pit houses in the southern Brazilian highlands. They are all later than the first millennium A.D., when the southern Jê groups were already established in the region for over a thousand years, and more than five centuries after the earliest documented pit house villages [1, 6, 20]. When these dates are considered together with our fine grained chronology for House 1, we can envisage the emergence of oversized pit houses to be a relatively recent phenomenon among the southern Jê groups, starting around A.D. 1000 and persisting in some regions until the early colonial era. Small pit houses continue to be inhabited during this time, which means that there is no replacement of one form of domestic architecture by another, but rather a process of emergent disparities in pit house size, even within the same settlements. We believe the rise of this phenomenon must be understood within a context of unprecedented landscape transformations, marked by an exponential increase in the number of domestic sites, intensification in the cultivation of domesticated plants, and appearance of other monumental expressions, represented by the ceremonial mound and enclosure complexes, at the time when palaeoecological records show the expansion of resource-rich *Araucaria* forests throughout the southern Brazilian highlands [11, 44].

**Table 2:** Radiocarbon dates for oversized pit houses in the southern Brazilian highlands. Levels marked with * correspond to the first occupation of the structures. Date Beta-178134 extends out of range of the calibration curve.

Site	House	House diameter (m)	House depth (m)	Stratum	Laboratory number	RCYBP	Cal A.D. 1σ	Cal A.D. 2σ	Reference
RS-AN-03	A	18	3.3	Layer 2b*	Beta-183020	880 ± 40	1160–1265	1050–1280	[21]
RS-AN-03	A	18	3.3	Layer 2a	Beta-183022	870 ± 50	1180–1270	1045–1285	[21]
RS-AN-03	A	18	3.3	Layer 2a	Beta-183021	690 ± 60	1290–1390	1265–1415	[21]
RS-AN-03	A	18	3.3	Layer 2	Beta-166584	370 ± 50	1495–1630	1455–1645	[21]
RS-AN-03	A	18	3.3	Layer 1	Beta-178134	250 ± 50	1635–1805	1505-present	[21]
SC-CL-52	N/N	20	7	Layer 6*	Beta-357350	860 ± 30	1205–1270	1175–1275	[5]

**Table 3:** Thermoluminescence dates for oversized pit houses in the southern Brazilian highlands. Dates are reported as in the original publication, where years BP refer to the date of the analysis (2001).

Site	House	House diameter (m)	House depth (m)	Stratum	Laboratory number	BP	A.D.	Reference
RS-A-27	3	14	2	Level 3	LVD-624	950 ± 72	1051	[2]
RS-A-27	3	14	2	Level 3	LVD-625	723 ± 55	1278	[2]

### Socio-cultural changes at Baggio I

**Fig 7:**
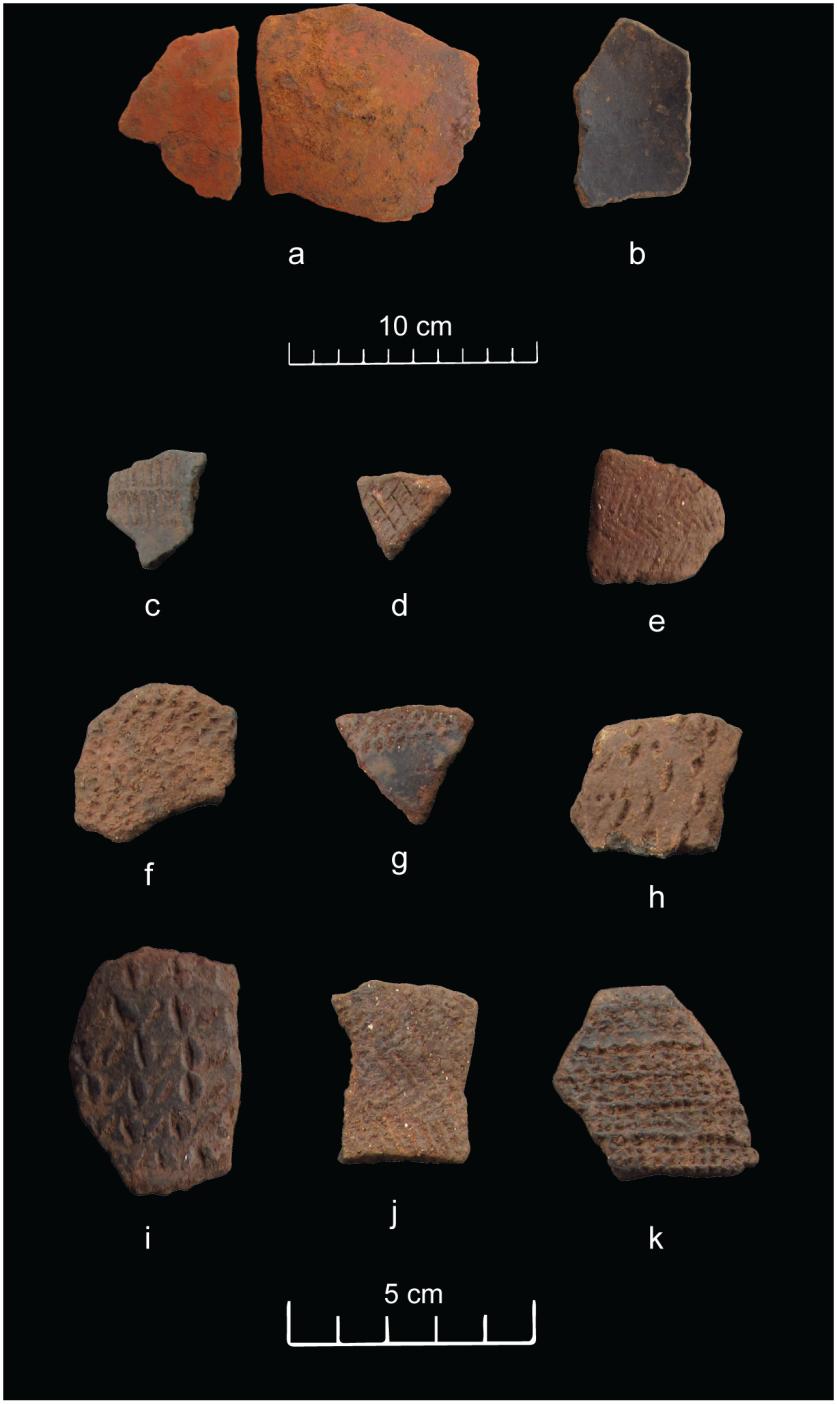
Surface finishing in ceramics from Baggio I. a) red slipped; b) black burnished; c-e) incised (parallel, cross-hatched, zigzag); f-i) punctate (punctate, fingernail impressed, finger pinched); j) basketry impression; k) stamped.

There were significant changes in material culture along over two centuries of occupation in House 1. For the first time, we could tie transformations in ceramic style and lithic technology to a detailed absolute chronology. Most of the ceramics from House 1 are identical in shape and decoration to the ceramics from the neighbouring areas, originally classified as the Xaxim, Guatambu and Guabiju phases [6]. One notable distinction in House 1 is the presence of a red slipped type (Fig 7a), which is particularly frequent in the burnt floor contexts (S1 and S2 Tables). Besides the surface finish, red slipped ceramics from House 1 are distinguished by being oxidised and much thicker than average, unlike the other ceramic types and Taquara-Itararé ceramics in general, which are well known for being thin and reduced (S3 Table) [6]. In most cases, the internal surface of the red slipped vessels are black burnished (Fig 7b) and frequently contains carbonised residue adhered to it, suggesting the use of red slipped ceramics for food processing (S3 Table).

**Fig 8:**
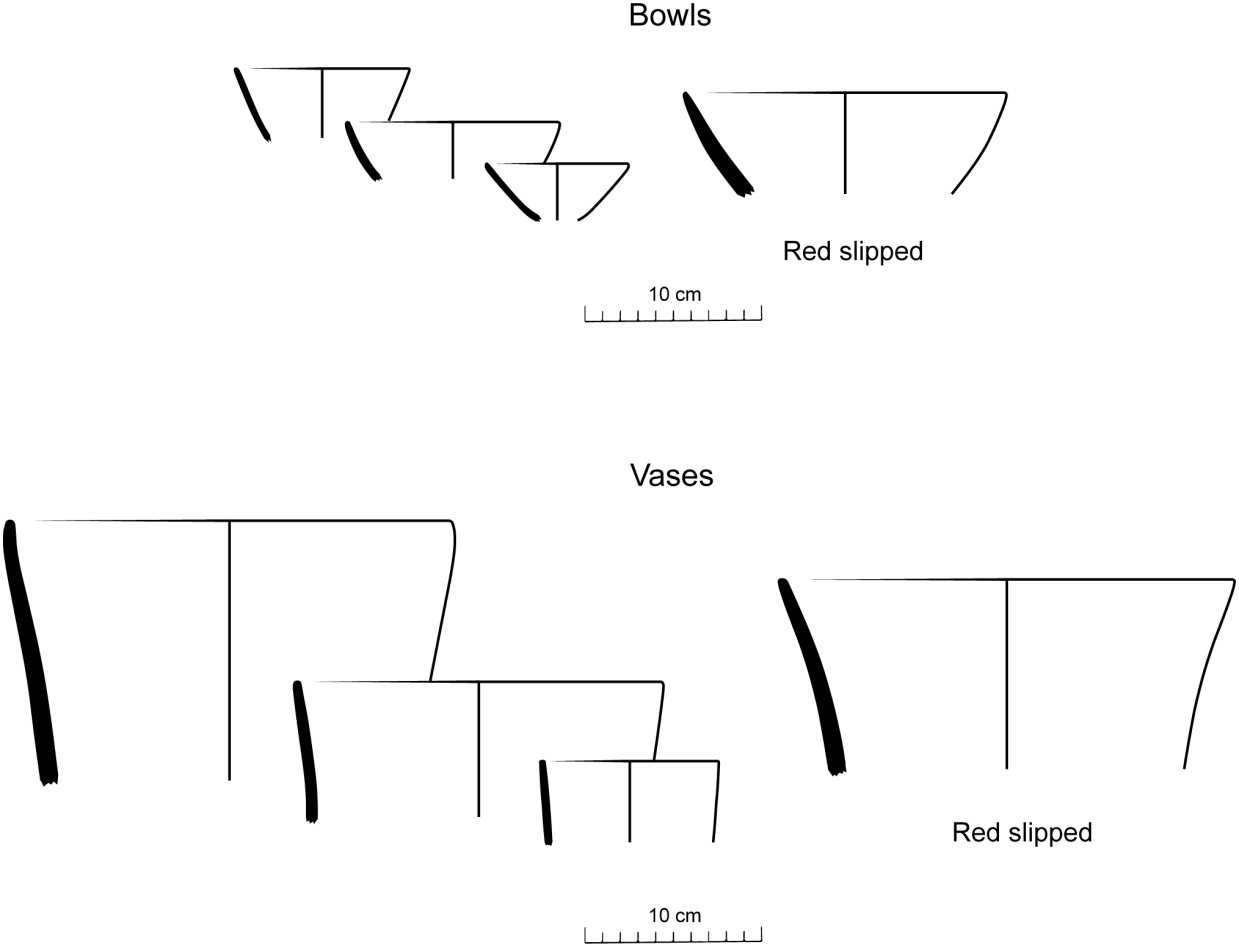
Reconstructed vessel shapes from House 1.

A large number of rims allowed the reconstruction of over 50 vessel profiles covering the entire sequence of House 1. The shapes are common to those reconstructed for the ceramic phases of the neighbouring areas [3, 6, 16]. Shallow hemispherical bowls and tall, cylindrical or conical vases with slight inflections are the dominant forms (Fig 8). The later can receive a band of plastic decoration close to the inflection point, and these decorated types include incised, punctate and stamped motifs (Fig 7c–7k). In House 1, ceramics with plastic decoration are very thin, extremely rare, and do not appear to have been used for cooking (S1–S3 Tables).

**Fig 9:**
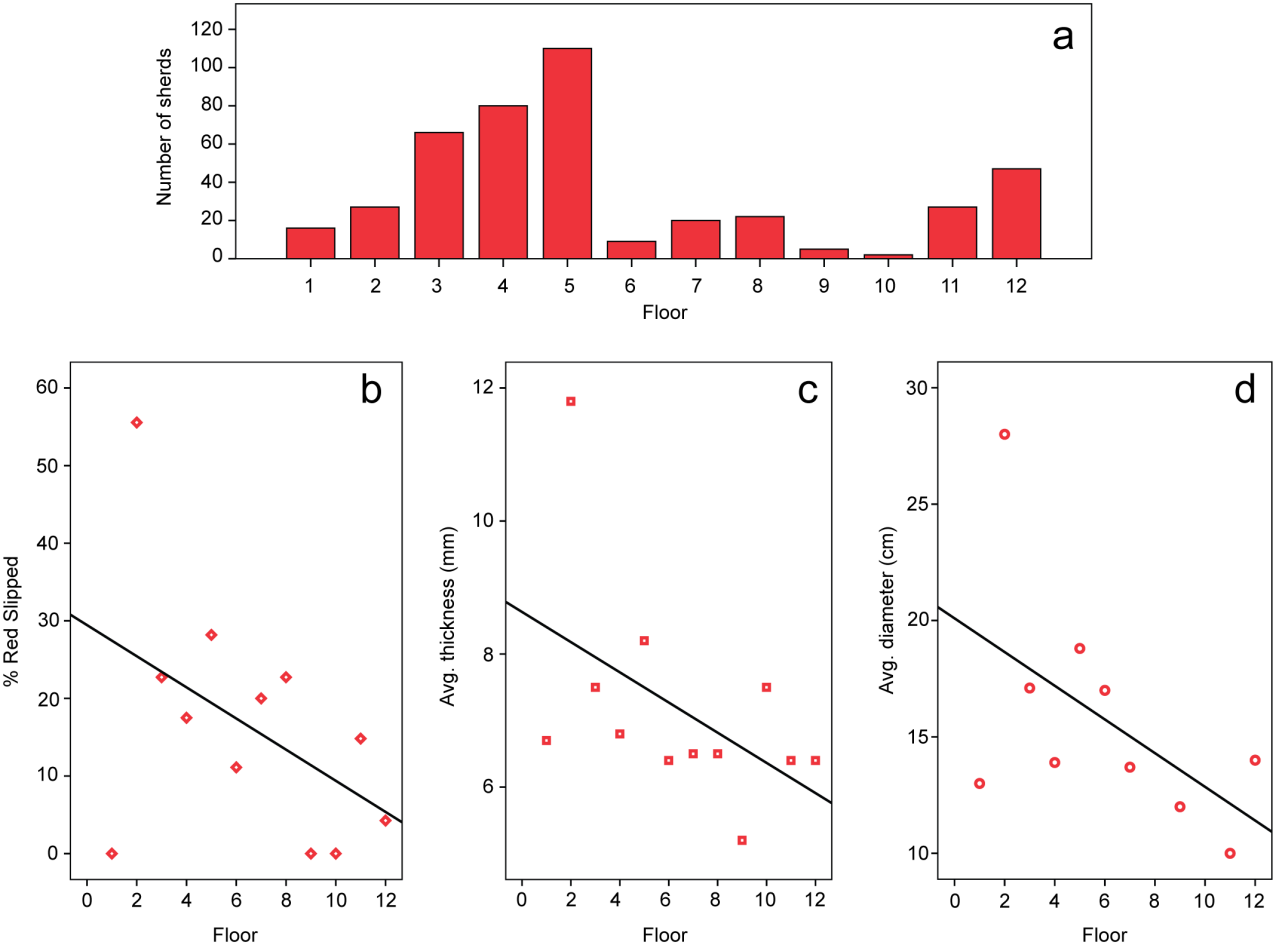
Change in the ceramics of House 1 over time. a) Quantity of ceramic sherds per floor; b) Percent of red slipped ceramic sherds per floor; c) Average sherd thickness per floor; d) Average vessel diameter per floor. Graphs b-d show linear regression line.

In terms of vertical distribution, the quantity of ceramics increases with each burnt floor until the last conflagration event (Fig 9a). In these contexts, ceramics lay directly on top of the charcoal layer, and therefore could not represent primary or *de facto* refuse, but must have been added after the collapse of the roof as part of termination events. We interpret the ceramic concentrations on top of the burnt floors as episodes of structured deposition [45], most likely dedicatory caches for the renewal of House 1 [46–48]. The most obvious case is the cache of large sherds, a small cup, and burnt bark on top of Floor 3 (Fig 5c).

Ceramics become rare in the later floors, as these surfaces were kept clean before subsequent construction events, but become frequent again in the last two floors before the terminal abandonment of House 1 (Fig 9a). There is a trend over time for a decreasing frequency of the red slipped type (Fig 9b). Although absent from the earliest floor, where, overall, ceramics were rare, this is the most abundant on Floor 2. A large proportion of the broken ceramics deposited on top of the subsequent burnt roofs belonged to the red slipped type, which gradually decreases in abundance in the later periods. Over time, ceramics also become thinner (Fig 9c) and smaller in diameter (Fig 9d). The last trend is not necessarily correlated with the decline of red slipped ceramics, since only two shapes were reconstructed for that type. We can conclude that ceramic vessels of all types tend to become considerably smaller by the end of the occupation of House 1.

In summary, for the earliest part of the history of House 1, the ceramic inventory included moderate to high proportions of the red slipped type. These were large vessels used in food processing and were discarded after each conflagration event. Red slipped ceramics are rare to absent in most southern proto-Jê domestic contexts [3, 5, 21], occurring more frequently in the northern limits of the tradition, far from our study area [6]. Closer to our study area, some oversized pit houses have been reported to contain a small number of red slipped ceramics, although never in the proportion found in Baggio I [2, 21]. We believe the large red ceramics from Baggio I might have constituted a special type of container used in the preparation and display of large quantities of food during conspicuous consumption events that were part of cyclical ceremonies preceding the destruction of the house by fire and its posterior entombment (for an Andean example, see [49]).

**Fig 10:**
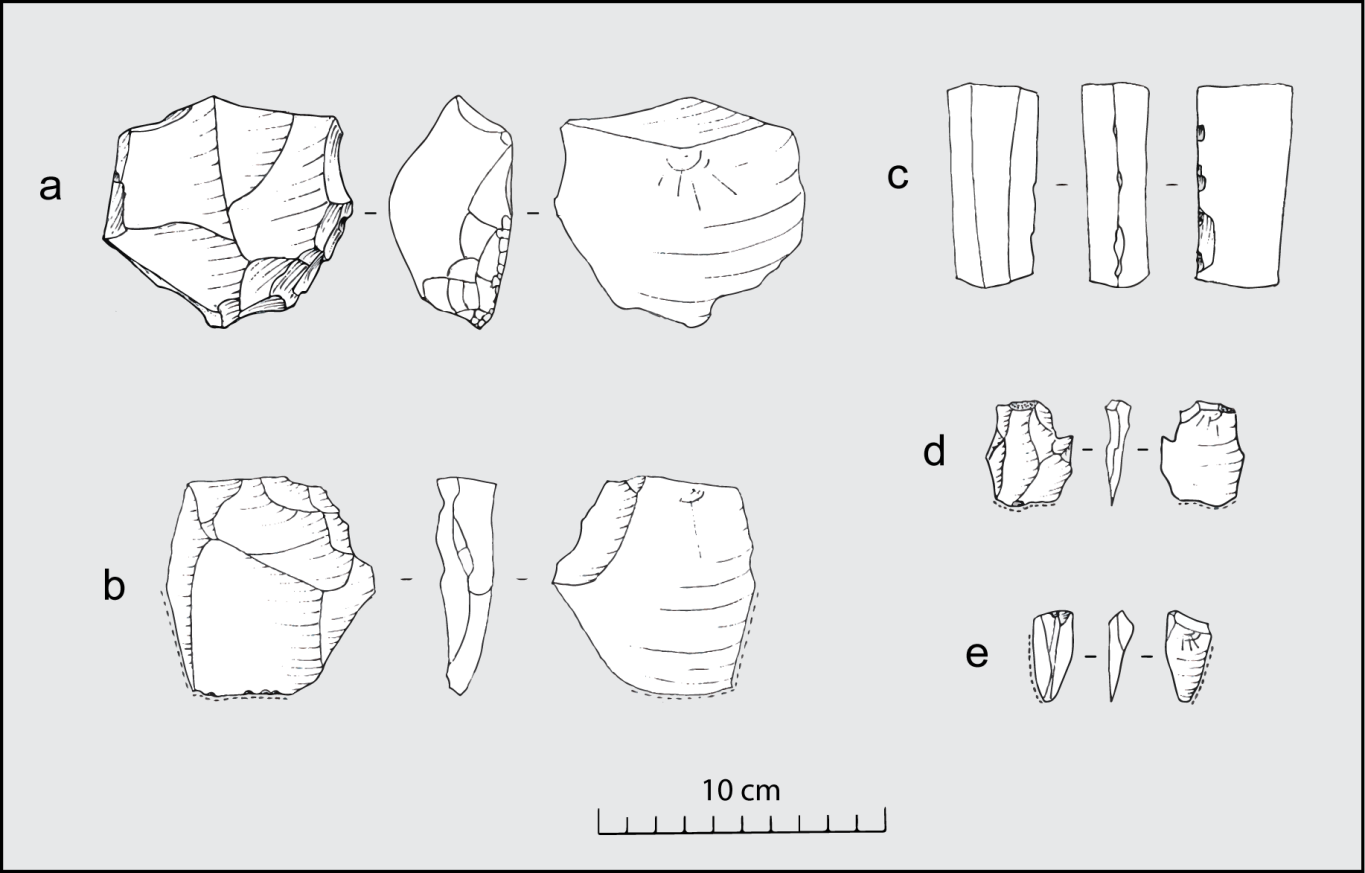
Lithics from Baggio I. a) Basalt scraper; b) Basalt flake with retouch and use wear; c) Piece of columnar basalt with retouch; d) Chert flake with use wear; e) Quartz flake with use wear.

**Fig 11:**
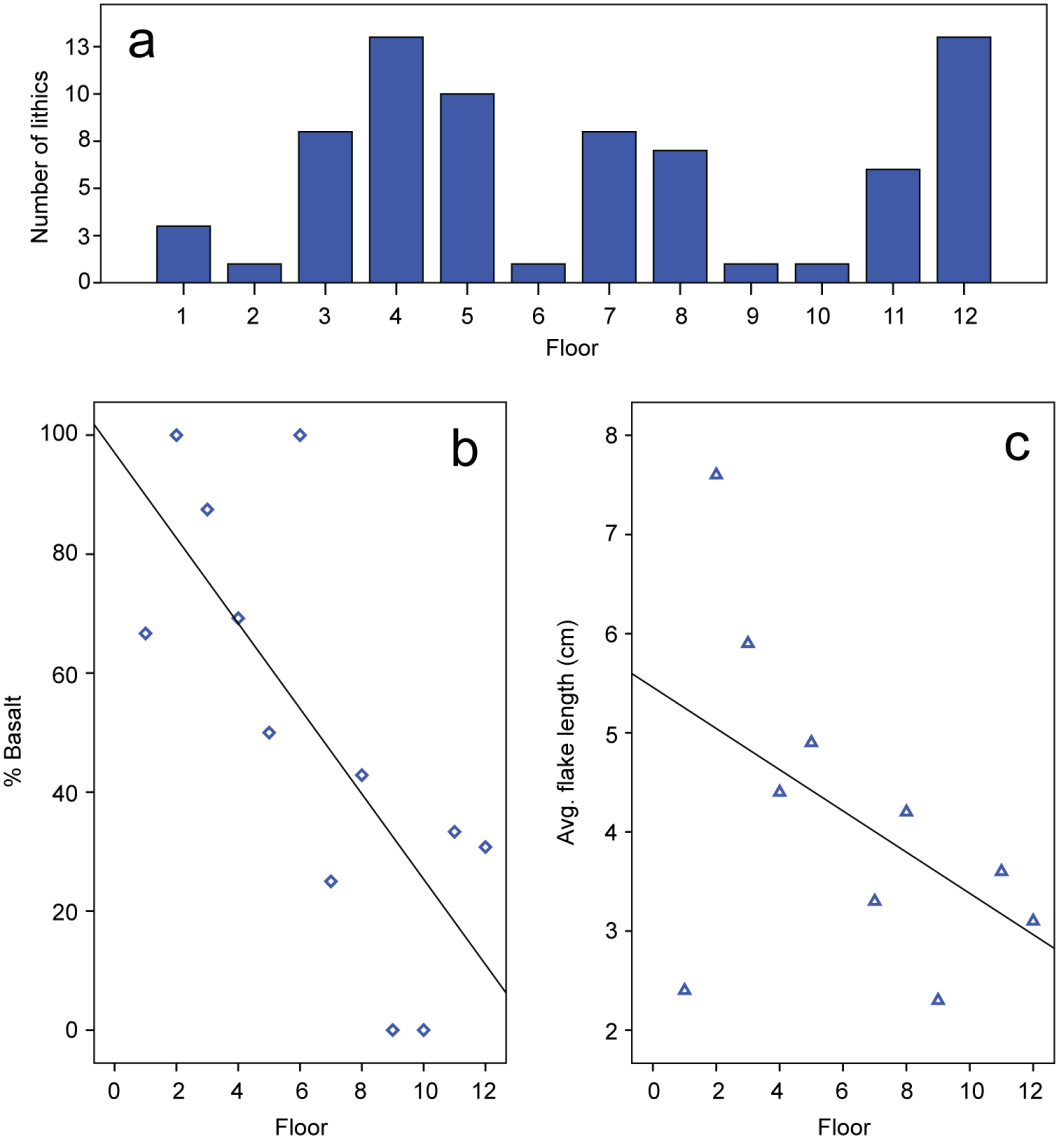
Change in the lithics of House 1 over time. a) Quantity of lithics per floor; b) Percent of basalt per floor; c) Average flake length per floor. Graphs b-c show linear regression line.

Lithics are few in number and do not exhibit major changes in assemblage composition through time (S4 and S5 Tables). Most of the material consists of *débitage*, with some flakes having been retouched and/or exhibiting use wear (Fig 10). As in the case of the ceramics, lithics are more abundant in the burnt floors than in the subsequent occupations, except for the period prior to abandonment (Fig 11a). The most noticeable trend is the gradual replacement of basalt as a favourite raw material by quartz and chert (Fig 11b, S6 Table) resulting in much smaller flakes during the later periods (Fig 11c). Lithic technology from the earliest floors of House 1 was directed towards the extraction of large basalt flakes that could be retouched (Fig 10b). On top of Floor 3, the lithic assemblage included a pair of columnar basalt pieces deposited side by side (Fig 10c). This predominance of large basalt flakes and tools (Fig 10a–10c) is later abandoned in favour of small chert and quartz flakes (Fig 10d and 10e).

### Occupation dynamics of House 1

Debates revolving around the degree of permanence in the pit houses of the southern Brazilian highlands are usually based on an insufficient number of radiocarbon dates [2, 16, 21]. The continuous occupation of House 1 for more than two centuries shows the importance of programmes of intensive dating of individual houses, and leads us to question assumptions of long periods of abandonment that are often based on isolated dates for pit houses selected from different sites [1, 2]. We believe that the discussions about regional population dynamics in the southern Brazilian highlands that are often based on few radiocarbon dates per site must be approached with extreme caution.

The dates from House 1 show a stable occupation enduring until the mid to late 17^th^ century (Table 1), proving that pit houses were still occupied in early colonial times, at least in the Canoas-Pelotas basin where the European presence was scarce until the 19^th^ century [50].

Furthermore, the sequence of floors in House 1 allows us to understand for the first time the history of activities at southern proto-Jê oversized pit houses. The initial period of occupation at the structure is marked by cyclical events of conflagration and entombment. Although completely burnt structures with impressive quantities of refuse are commonly interpreted as resulting from accidental or warfare events [51], they might in fact represent ritual modes of architectural abandonment and renewal [52]. The deliberate burning of domestic and ceremonial structures is common worldwide as part of rituals of termination and renewal [46, 47, 53–56]. The repetitive pattern found in the first five floors of House 1 points to intentional destruction by fire, followed by the covering of the collapsed burnt roof with thick layers of up to 20 cm of sterile clay to create a new floor surface. This practice resembles the process of entombment—the intentional filling of domestic or ceremonial buildings before their expansion or the construction of new structures directly on top [49, 56–58]. This process, aptly called “stratigraphy-making” by McAnany and Hodder [56], is often linked to cyclical or calendric rituals of renewal [58, 59]. The repetitious pattern of occupation, collapsing and burning of the roof, breaking of pottery and deposition of ceremonial caches, intercalated with fill strata, reveals a social tempo or rhythm in household ritual that can be related to prolonged social calendars and ordered ritual stages [35] so far never recorded in the southern proto-Jê contexts.

Interestingly, the interval between each floor of House 1 is not constant. As can be inferred from the intervals between the modelled dates (Table 1, Fig 6), the earliest floors, where the practice of burning the roof was documented, appear to have been resurfaced after a longer time span. The earliest three floors have dates whose probability distributions exhibit little to no overlap, their medians separated in average by 60–65 years, whereas the subsequent floors have dates that are very close, with a great deal of overlap even between the modelled distributions, and an average distance of 15–30 years between their medians (Table 1, Fig 6). One possible interpretation is that the potentially longer cycles of conflagration ceremonies during the first part of the history of House 1 were most likely performed after the death of a prominent member of the community, maybe the chief of an extended family living in the pit house. The burning down of houses after an occupant’s death, followed by its abandonment, is a widely documented practice [52]. Interestingly, this ritual practice was intensified in House 1 after Floor 3 (Cal. A.D. 1485–1550), with an increasing amount of goods being interred with the burnt house in shorter intervals of time.

We can tentatively link this intensification in ritual burning, as well as the abandonment of this practice, to the indirect impact of the European presence. Studies demonstrating similar changes at the turn of the 16^th^ century are still rare: on the adjacent coast of Santa Catarina, Milheira [60] has excavated settlements of the Guarani archaeological tradition with burnt houses—although, in this case, they contained complete usable domestic toolkits, suggesting a rapid abandonment. The dates, between 440 ± 40 ^14^C BP (Cal. A.D. 2σ 1430–1625) and 430 ± 40 ^14^C BP (Cal. A.D. 2σ 1435–1630), led him to associate the conflagration episodes with increased violence brought about by the Portuguese presence on the coast [60]. However, the direct impact of the European contact would not have been felt in the core of the highlands until the 17^th^ century. This means that, if the changes observed in House 1 at the turn of the 16^th^ century can be attributed to the arrival of the Portuguese, it must have been through indirect effects (such as diseases or the pushing of other groups to the interior).

The rituals of conflagration, structured deposition and entombment at House 1 were no longer practiced after Floor 5 (Cal. A.D. 1510–1585). Instead, regular maintenance of the house kept the floors clean of most debris, with the exception of a few small ceramic sherds and lithics that escaped sweeping and were trampled into the floor, being incorporated into the matrix after resurfacing events. This interval likely represents more mundane concerns with house maintenance than the long cycles of the earliest floors. The last floor of House 1, dated Cal. A.D. 1625–1685, contained small fire pits and a significant quantity of primary refuse, being the result of the definitive abandonment of the house. The small-sized primary debris left to accumulate on the floor around the fire pits, coupled with the absence of refuse with large dimensions, valuable items, caches of still usable artefacts, or even provisionally discarded ones appears to point to a scenario of gradual abandonment, but with no expectation of return to the site [61, 62].

Thus, even though ritual burning was no longer practiced at House 1 after Floor 5 (Cal. A.D. 1510–1585), the house remained in use until its terminal abandonment with no major chronological gap and no abrupt change in material culture, suggesting that the same lineage kept occupying the structure. The fact that the house was not avoided, with new floors superimposed on old ones, shows a sense of memory and the concern of its occupants for marking their continuity with past generations [56].

In the same way as the lavish displays involved in burning the house and filling it with ceremonial deposits are abandoned, changes in material culture soon follow: the large, red slipped vessels that accompanied the burnt floors were gradually replaced by smaller, thinner ceramics. When seen in conjunction with the features from the later floors, mostly consisting of very small fire pits, this is suggestive of a transition to more intimate practices, involving food preparation and consumption by and for smaller groups of individuals. Probably the communal bonds of an early extended family household were broken into their nuclear family constituents. If that is the case, the hypothesis of a transition from extended to nuclear families [23], if true, would not imply the abandonment of the original oversized dwelling. The house would have been maintained by the later occupants as a materialisation of social memory that ensured their connection to the founders of the site [56]. In summary, all the evidence from House 1 strongly supports the existence of long-lived residential corporate groups [63, 64] for the first time in the archaeology of the southern Brazilian highlands.

## Conclusions

Our programme of comprehensive dating of House 1, with the subsequent Bayesian modelling of the radiocarbon dates, provided the first solid stratigraphic and chronological evidence for continuous occupation of an oversized pit house in the southern Brazilian highlands. We have demonstrated that House 1 was occupied for for over two centuries with no major hiatus, contradicting previous models that postulated that the pit house villages of the southern proto-Jê groups were the result of long cycles of abandonment and short-term reoccupations.

During this long-term occupation of House 1, we could observe changes in the construction processes, pace of architectural renovation, ceramic styles and lithic technology. The chronology obtained with Bayesian statistics allowed us to tie these transformations in household practices with a long sequence of absolute dates. Our results show the importance of programmes of intensive dating of single house structures in order to understand the degree of permanence and changes in occupation dynamics of pit house villages.

Throughout the history of House 1, stratigraphy demonstrates that the structure was never abandoned, but constantly renewed, showing that its occupants were concerned with marking their continuous presence by preparing new floors on top of the old ones. The modelled dates show that this practice is continuous, without any large hiatus. This points to the possibility that members of a single lineage or corporate group occupied the structure throughout its history: they were passing through major transformations in architectural renovation practices, ceramic style and lithic technology, whilst assertively marking their presence in a single domestic structure for over two centuries.

Our results add to the increasing amount of evidence that is beginning to offer a different picture of the southern proto-Jê groups. It is now clear that a moderate degree of social inequality was present among those groups, and that land use strategies were more intense than previously thought. Renewed research on the mound and enclosure complexes has revealed a complex monumental ritual landscape with differential mortuary treatment for few individuals [11, 14, 18]. Recent archaeobotanical analysis has shown that a range of cultigens were present in pit house contexts, including cultivated products from all seasons, contradicting previous views that postulated a high degree of mobility among those groups and an incipient horticulture practiced for only part of the year [3]. Finally, our conclusion that House 1 attests the existence of long-lived residential corporate groups [63, 64] with elaborate rituals of architectural renewal and a high degree of site permanence has major implications for the understanding of the socio-political organisation of the southern proto-Jê groups, challenging the view, dominant until relatively recently, that these were marginal cultures in the context of lowland South America.

## Supporting Information

S1 Appendix OxCal model specification. (PDF)

S1 Table Ceramic data from House 1. (PDF)

S2 Table Ceramic types per floor in House 1. (PDF)

S3 Table Attributes of the ceramic types identified in House 1. (PDF)

S4 Table Lithic data from House 1. (PDF)

S5 Table Lithic assemblage for each floor of House 1. (PDF)

S6 Table Lithic raw material per floor in House 1. (PDF)
