# Supplementary material for: Understanding the Chronology and Occupation Dynamics of Oversized Pit Houses in the Southern Brazilian Highlands
Source: PLoS One. 2016 Jul 6;11(7):e0158127. doi: 10.1371/journal.pone.0158127 (PMC4934860; doi:10.1371/journal.pone.0158127)
Supplement: S1 Appendix — (PDF) [file pone.0158127.s001.pdf]

```
Plot()
    Curve("ShCal13","ShCal13.14c")
    Sequence("Baggio 1, House 1")
        Boundary("Start House 1")
        Phase("Floor 1")
            R_Date("Beta 414089", 630, 30)

        Boundary("Transition 1/2")
        Phase("Floor 2")
            R_Date("Beta 414088", 460, 30)

        Boundary("Transition 2/3")
        Phase("Floor 3")
            R_Date("Beta 414087", 300, 30)

        Boundary("Transition 3/4")
        Phase("Floor 4")
            R_Date("Beta 414086", 860,30)
                Outlier()


        Boundary("Transition 4/5")
        Phase("Floor 5")
            R_Date("Beta 414085", 340, 30)

        Boundary("Transition 5/7")
        Phase("Floor 7")
            R_Date("Beta 414084", 350, 30)

        Boundary("Transition 7/8")
        Phase("Floor 8")
            R_Date("Beta 414083", 520, 30)
                Outlier()


        Boundary("Transition 8/9")
        Phase("Floor 9")
            R_Date("Beta 414091", 360, 30)

        Boundary("Transition 9/10")
        Phase("Floor 10")
            R_Date("Beta 414082", 350, 30)

        Boundary("Transition 10/11")
        Phase("Floor 11")
            R_Date("Beta 414081", 340, 30)

        Boundary("Transition 11/12")
        Phase("Floor 12")
            R_Date("Beta 414080", 280, 30)

        Boundary("End House 1")
```

Parameters

Parameter	Name	Type	Functions	z_i	μ_i	σ_i	$llim_i$	$ulim_i$
$\mathbf{r}_1$, $\mathbf{s}_1$	intcal13	Curve					-48054.5	1965.5
$\mathbf{r}_2$, $\mathbf{s}_2$	ShCal13	Curve					-48054.5	1965.5
t_3	Start House 1	Boundary			1375.34	33.7207	860.5	1465.5
t_4	Beta 414089	R_Date			1392.52	23.0165	1260.5	1465.5
t_5	Transition 1/2	Boundary			1423.5	28.9853	1260.5	1660.5
t_6	Beta 414088	R_Date			1461.95	16.1542	1380.5	1660.5
t_7	Transition 2/3	Boundary			1495.23	22.642	1380.5	1810.5
t_8	Beta 414087	R_Date			1518.31	13.8311	1440.5	1810.5
t_9	Transition 3/4	Boundary			1527.3	16.0437	1440.5	1810.5
t_{10}	Beta 414086	R_Date			1226.79	27.8109	1015.5	1395.5
t_{11}	Transition 4/5	Boundary			1541.05	17.9083	1440.5	1810.5
t_{12}	Beta 414085	R_Date			1548.06	18.2025	1440.5	1810.5
t_{13}	Transition 5/7	Boundary			1555.28	19.4999	1440.5	1810.5
t_{14}	Beta 414084	R_Date			1562.71	19.9493	1440.5	1810.5
t_{15}	Transition 7/8	Boundary			1570.71	21.5131	1440.5	1810.5
t_{16}	Beta 414083	R_Date			1432.76	13.0176	1300.5	1635.5
t_{17}	Transition 8/9	Boundary			1586.79	22.8537	1440.5	1810.5
t_{18}	Beta 414091	R_Date			1594.83	22.5138	1440.5	1810.5
t_{19}	Transition 9/10	Boundary			1603.22	22.3753	1440.5	1815.5
t_{20}	Beta 414082	R_Date			1611.59	21.0168	1440.5	1815.5
t_{21}	Transition 10/11	Boundary			1619.79	19.7278	1440.5	1815.5
t_{22}	Beta 414081	R_Date			1628.4	17.2556	1440.5	1815.5
t_{23}	Transition 11/12	Boundary			1638	18.3416	1440.5	1965.5
t_{24}	Beta 414080	R_Date			1648.53	19.1728	1440.5	1965.5
t_{25}	End House 1	Boundary			1661.8	28.6764	1440.5	2490.5

Likelihoods

$p(y_i\|t_j)$	F_i		A_i=100 F_i
$p(y_4\|t_4)$ ~ R_Date$_4$(t_4, $\mathbf{r}_2$, $\mathbf{s}_2$, 630,30)	$F_4 = (\int p(y_4\|t_4)\, p(t_4\|\mathbf{y})\, dt_4)/(\int p^2(y_4\|t_4)\, dt_4)$	0.86950	86.95
$p(y_6\|t_6)$ ~ R_Date$_6$(t_6, $\mathbf{r}_2$, $\mathbf{s}_2$, 460,30)	$F_6 = (\int p(y_6\|t_6)\, p(t_6\|\mathbf{y})\, dt_6)/(\int p^2(y_6\|t_6)\, dt_6)$	1.10100	110.10
$p(y_8\|t_8)$ ~ R_Date$_8$(t_8, $\mathbf{r}_2$, $\mathbf{s}_2$, 300,30)	$F_8 = (\int p(y_8\|t_8)\, p(t_8\|\mathbf{y})\, dt_8)/(\int p^2(y_8\|t_8)\, dt_8)$	0.57410	57.41
$p(y_{10}\|t_{10})$ ~ R_Date$_{10}$(t_{10}, $\mathbf{r}_2$, $\mathbf{s}_2$, 860,30)	$F_{10} = (\int p(y_{10}\|t_{10})\, p(t_{10}\|\mathbf{y})\, dt_{10})/(\int p^2(y_{10}\|t_{10})\, dt_{10})$		
$p(y_{12}\|t_{12})$ ~ R_Date$_{12}$(t_{12}, $\mathbf{r}_2$, $\mathbf{s}_2$, 340,30)	$F_{12} = (\int p(y_{12}\|t_{12})\, p(t_{12}\|\mathbf{y})\, dt_{12})/(\int p^2(y_{12}\|t_{12})\, dt_{12})$	1.14390	114.39
$p(y_{14}\|t_{14})$ ~ R_Date$_{14}$(t_{14}, $\mathbf{r}_2$, $\mathbf{s}_2$, 350,30)	$F_{14} = (\int p(y_{14}\|t_{14})\, p(t_{14}\|\mathbf{y})\, dt_{14})/(\int p^2(y_{14}\|t_{14})\, dt_{14})$	1.11520	111.52
$p(y_{16}\|t_{16})$ ~ R_Date$_{16}$(t_{16}, $\mathbf{r}_2$, $\mathbf{s}_2$, 520,30)	$F_{16} = (\int p(y_{16}\|t_{16})\, p(t_{16}\|\mathbf{y})\, dt_{16})/(\int p^2(y_{16}\|t_{16})\, dt_{16})$		
$p(y_{18}\|t_{18})$ ~ R_Date$_{18}$(t_{18}, $\mathbf{r}_2$, $\mathbf{s}_2$, 360,30)	$F_{18} = (\int p(y_{18}\|t_{18})\, p(t_{18}\|\mathbf{y})\, dt_{18})/(\int p^2(y_{18}\|t_{18})\, dt_{18})$	1.01190	101.19
$p(y_{20}\|t_{20})$ ~ R_Date$_{20}$(t_{20}, $\mathbf{r}_2$, $\mathbf{s}_2$, 350,30)	$F_{20} = (\int p(y_{20}\|t_{20})\, p(t_{20}\|\mathbf{y})\, dt_{20})/(\int p^2(y_{20}\|t_{20})\, dt_{20})$	0.93600	93.60
$p(y_{22}\|t_{22})$ ~ R_Date$_{22}$(t_{22}, $\mathbf{r}_2$, $\mathbf{s}_2$, 340,30)	$F_{22} = (\int p(y_{22}\|t_{22})\, p(t_{22}\|\mathbf{y})\, dt_{22})/(\int p^2(y_{22}\|t_{22})\, dt_{22})$	0.96630	96.63
$p(y_{24}\|t_{24})$ ~ R_Date$_{24}$(t_{24}, $\mathbf{r}_2$, $\mathbf{s}_2$, 280,30)	$F_{24} = (\int p(y_{24}\|t_{24})\, p(t_{24}\|\mathbf{y})\, dt_{24})/(\int p^2(y_{24}\|t_{24})\, dt_{24})$	1.43240	143.24
**n**		9	
$\mathbf{F_{model}}$	$\int p(\mathbf{y}\|\mathbf{t})\, p(\mathbf{t}\|\mathbf{y})\, dt \,/\, \int p(\mathbf{y}\|\mathbf{t})\, p(\mathbf{y}\|\mathbf{t})\, dt$	0.903286	
$\mathbf{A_{model}}$	$100\, F_{model}^{1/\sqrt{n}}$	96.67	
$\mathbf{F_{overall}}$	$\prod F_i$	0.919076	
$\mathbf{A_{overall}}$	$100\, F_{overall}^{1/\sqrt{n}}$	97.23	

Constraints				
$t_3 < t_4$	$t_4 < t_5$	$t_5 < t_6$	$t_6 < t_7$	$t_7 < t_8$
$t_8 < t_9$	t_9 ?< t_{10}	$t_9 < t_{11}$	t_{10} ?< t_{11}	$t_{11} < t_{12}$
$t_{12} < t_{13}$	$t_{13} < t_{14}$	$t_{14} < t_{15}$	t_{15} ?< t_{16}	$t_{15} < t_{17}$
t_{16} ?< t_{17}	$t_{17} < t_{18}$	$t_{18} < t_{19}$	$t_{19} < t_{20}$	$t_{20} < t_{21}$
$t_{21} < t_{22}$	$t_{22} < t_{23}$	$t_{23} < t_{24}$	$t_{24} < t_{25}$	

Prior factors
$1/\min((ulim_{25} - llim_3) - (t_{25} - t_3),(t_{25} - t_3) - (llim_{25} - ulim_3),ulim_{25} - llim_{25},ulim_3 - llim_3)$
$1/(t_5 - t_3)^1$
$1/(t_7 - t_5)^1$
$1/(t_9 - t_7)^1$
$1/(t_{13} - t_{11})^1$
$1/(t_{15} - t_{13})^1$
$1/(t_{19} - t_{17})^1$
$1/(t_{21} - t_{19})^1$
$1/(t_{23} - t_{21})^1$
$1/(t_{25} - t_{23})^1$
$1/(t_{25} - t_3)^{10}$

Groupings used in generating above priors

Type	b_{lower}	type	elements	n	b_{upper}	type
Sequence	t_3	Boundary	t_4	1	t_5	Boundary
Sequence	t_5	Boundary	t_6	1	t_7	Boundary
Sequence	t_7	Boundary	t_8	1	t_9	Boundary
Sequence	t_9	Boundary		0	t_{11}	Boundary
Sequence	t_{11}	Boundary	t_{12}	1	t_{13}	Boundary
Sequence	t_{13}	Boundary	t_{14}	1	t_{15}	Boundary
Sequence	t_{15}	Boundary		0	t_{17}	Boundary
Sequence	t_{17}	Boundary	t_{18}	1	t_{19}	Boundary
Sequence	t_{19}	Boundary	t_{20}	1	t_{21}	Boundary
Sequence	t_{21}	Boundary	t_{22}	1	t_{23}	Boundary
Sequence	t_{23}	Boundary	t_{24}	1	t_{25}	Boundary
Sequence	t_3	Boundary	t_5, t_7, t_9, t_{11}, t_{13}, t_{15}, t_{17}, t_{19}, t_{21}, t_{23}	10	t_{25}	Boundary
