# Supplementary material for: Understanding the Chronology and Occupation Dynamics of Oversized Pit Houses in the Southern Brazilian Highlands
Source: PLoS One. 2016 Jul 6;11(7):e0158127. doi: 10.1371/journal.pone.0158127 (PMC4934860; doi:10.1371/journal.pone.0158127)
Supplement: S1 Table — (PDF) [file pone.0158127.s002.pdf]

Unit	Stratum	Find #	Class	Rim Ø (cm)	Burning	Thickness (mm)	External surface	Internal surface
Area A	Floor 12	27	Rim	18	Reduced	9	Polished	Polished
Area A 100/104	Floor 12	22	Wall		Reduced	5	Polished	Polished
Area A 100/104	Floor 12	23	Wall		Reduced	6	Black burnished	Polished
Area A 100/104	Floor 12	24	Wall		Reduced	9	Polished	Polished
Area A 100/104	Floor 12	26	Wall		Reduced	4	Black burnished	Black burnished
Area A 100/104	Floor 12	52	Infl. point		Reduced	6	Polished	Red slipped
Area A 100/105	Floor 12	21	Rim	12	Reduced	5	Eroded	Eroded
Area A 100/105	Floor 12	24	Wall		Reduced	5	Nail impressed	Polished
Area A 100/105	Floor 12	25	Wall		Partly ox.	5	Polished	Black burnished
Area A 100/105	Floor 12	-	Wall		Reduced	5	Red slipped	Black burnished
Area A 100/105	Floor 12	-	Wall		Partly ox.	6	Polished	Polished
Area A 100/105	Floor 12	-	Wall		Reduced	7	Incised	Polished
Area A 101/104	Floor 12	17	Wall		Partly ox.	10	Eroded	Polished
Area A 101/104	Floor 12	-	Wall		Reduced	5	Nail impressed	Polished
Area A 101/105	Floor 12	22	Wall		Reduced	4	Polished	Polished
Area A 101/105	Floor 12	24	Wall		Reduced	8	Polished	Polished
Area A 101/105	Floor 12	24	Wall		Reduced	6	Polished	Polished
Area A 101/105	Floor 12	26	Wall		Reduced	5	Incised	Black burnished
Area A 101/105	Floor 12		Wall		Reduced	6	Black burnished	Polished
Area B 100/107	Floor 12		Wall		Oxyd.	4	Black burnished	Eroded
Area B 100/107	Floor 12	34	Wall		Reduced	4	Polished	Polished
Area B 100/107	Floor 12	35	Wall		Reduced	5	Polished	Black burnished
Area B 100/107	Floor 12	36	Wall		Reduced	6	Eroded	Eroded
Area B 100/107	Floor 12	40	Wall		Reduced	7	Eroded	Polished
Area B 100/107	Floor 12	41	Wall		Reduced	6	Eroded	Black burnished
Area B 100/107	Floor 12	49	Wall		Reduced	6	Eroded	Eroded
Area B 100/107	Floor 12	51	Wall		Reduced	9	Polished	Polished
Area B 100/107	Floor 12	53	Wall		Oxyd.	4	Red slipped	Red slipped
Area B 100/107	Floor 12	54	Wall		Reduced	6	Polished	Polished
Area B 100/107	Floor 12	56	Wall		Reduced	5	Eroded	Eroded
Area B 100/107	Floor 12	57	Wall		Partly ox.	10	Polished	Black burnished
Area B 100/108	Floor 12	22	Wall		Reduced	6	Black burnished	Black burnished
Area B 100/108	Floor 12	23	Base		Reduced	8	Eroded	Eroded
Area B 100/108	Floor 12	24	Wall		Reduced	7	Polished	Polished
Area B 100/108	Floor 12	25	Wall		Reduced	5	Black burnished	Black burnished
Area B 100/108	Floor 12	26	Wall		Reduced	5	Polished	Polished
Area B 101/107	Floor 12	30	Wall		Partly ox.	8	Eroded	Eroded
Area B 101/107	Floor 12	31	Wall		Partly ox.	8	Eroded	Eroded
Area B 101/107	Floor 12	32	Wall		Reduced	7	Polished	Black burnished
Area B 101/107	Floor 12	33	Wall		Partly ox.	8	Eroded	Eroded
Area B 101/107	Floor 12	42	Wall		Reduced	8	Black burnished	Black burnished
Area B 101/107	Floor 12	44	Wall		Oxyd.	10	Eroded	Red slipped
Area B 101/108	Floor 12	10	Wall		Reduced	5	Black burnished	Polished
Area B 101/108	Floor 12	12	Wall		Reduced	6	Eroded	Eroded
Area B 101/108	Floor 12	8	Wall		Reduced	9	Eroded	Polished
Area B 101/108	Floor 12	9	Wall		Partly ox.	5	Polished	Polished
Area B 101/108	Floor 12	-	Wall		Partly ox.	6	Polished	Polished
Area A 100/104	Floor 11	29	Infl. point		Partly ox.	6	Pinched	Polished

Area A 100/104	Floor 11	32	Wall		Reduced	5	Eroded	Eroded
Area A 100/104	Floor 11	33	Wall		Reduced	6	Eroded	Black burnished
Area A 100/104	Floor 11	28	Wall		Oxyd.	5	Black burnished	Black burnished
Area A 100/104	Floor 11	-	Wall		Partly ox.	9	Red slipped	Black burnished
Area A 100/105	Floor 11	29	Wall		Reduced	6	Polished	Eroded
Area A 100/105	Floor 11	33	Wall		Reduced	4	Polished	Polished
Area A 101/104	Floor 11	28	Wall		Reduced	6	Eroded	Eroded
Area A 101/104	Floor 11	20	Wall		Reduced	5	Polished	Polished
Area A 101/104	Floor 11	21	Wall		Reduced	5	Red slipped	Polished
Area A 101/104	Floor 11	22	Wall		Reduced	11	Polished	Polished
Area A 101/104	Floor 11	24	Wall		Partly ox.	6	Polished	Polished
Area A 101/105	Floor 11	29	Wall		Reduced	4	Black burnished	Polished
Area A 101/105	Floor 11	32	Wall		Reduced	5	Eroded	Eroded
Area A 101/105	Floor 11	33	Infl. point		Reduced	9	Polished	Polished
Area B 100/107	Floor 11	63	Wall		Reduced	6	Polished	Polished
Area B 100/107	Floor 11	65	Wall		Reduced	5	Polished	Polished
Area B 100/107	Floor 11	60	Wall		Reduced	7	Eroded	Eroded
Area B 100/107	Floor 11	61	Wall		Reduced	7	Polished	Polished
Area B 100/108	Floor 11	30	Wall		Reduced	4	Eroded	Polished
Area B 101/107	Floor 11	63	Wall		Partly ox.	5	Polished	Polished
Area B 101/107	Floor 11	8			Partly ox.	9	Red slipped	Polished
Area B 101/107	Floor 11	5	Wall		Partly ox.	6	Eroded	Eroded
Area B 101/107	Floor 11	54	Wall		Partly ox.	12	Red slipped	Black burnished
Area B 101/107	Floor 11	55	Wall		Reduced	5	Polished	Polished
Area B 101/107	Floor 11	58	Wall		Partly ox.	5	Polished	Polished
Area B 101/108	Floor 11	13	Wall		Reduced	10	Polished	Polished
Area A 101/105	Floor 10	36	Wall		Reduced	8	Eroded	Eroded
Area A 101/105	Floor 10	37	Wall		Reduced	7	Polished	Black burnished
Area B 100/108	Floor 9	33	Wall		Reduced	5	Black burnished	Black burnished
Area B 100/108	Floor 9	36	Wall		Reduced	6	Polished	Polished
Area B 101/107	Floor 9	-	Wall		Reduced	4	Polished	Polished
Area B 101/108	Floor 9	17	Wall		Reduced	3	Polished	Polished
Area B 101/108	Floor 9	18	Wall		Reduced	8	Black burnished	Black burnished
Area A	Floor 8	-	Wall		Reduced	9	Red slipped	Black burnished
Area A	Floor 8	-	Wall		Reduced	5	Polished	Polished
Area A	Floor 8	65	Wall		Reduced	8	Red slipped	Black burnished
Area A	Floor 8	-	Wall		Reduced	4	Polished	Polished
Area A	Floor 8	-	Wall		Oxyd.	5	Polished	Polished
Area A	Floor 8	-	Wall		Reduced	6	Polished	Polished
Area A	Floor 8	-	Rim	12	Reduced	6	Black burnished	Polished
Area A 100/104	Floor 8	41	Wall		Reduced	5	Black burnished	Black burnished
Area A 100/104	Floor 8	43	Wall		Partly ox.	5	Stamped	Eroded
Area A 100/104	Floor 8	48	Rim	32	Reduced	7	Polished	Polished
Area A 100/104	Floor 8	57	Wall		Partly ox.	7	Red slipped	Eroded
Area A 100/104	Floor 8	58	Wall		Partly ox.	9	Red slipped	Polished
Area A 100/104	Floor 8	61	Rim		Reduced	9	Polished	Polished
Area A 100/105	Floor 8	49	Wall		Reduced	5	Eroded	Eroded
Area A 100/105	Floor 8	52	Wall		Reduced	8	Polished	Polished
Area A 100/105	Floor 8	63	Wall		Reduced	9	Red slipped	Black burnished
Area A 101/105	Floor 8	37	Wall		Partly ox.	7	Eroded	Eroded

Area A 101/105	Floor 8	51	Wall		Reduced	6	Polished	Polished
Area A 101/105	Floor 8	51	Wall		Reduced	5	Polished	Polished
Area A 101/105	Floor 8	56	Wall		Reduced	7	Black burnished	Black burnished
Area B 100/107	Floor 8	70	Wall		Reduced	7	Polished	Polished
Area B 101/107	Floor 8	69	Wall		Reduced	5	Polished	Polished
Area A	Floor 7	77	Wall		Oxyd.	5	Polished	Polished
Area A	Floor 7	78	Wall		Oxyd.	5	Eroded	Black burnished
Area A	Floor 7	81	Infl. point		Reduced	6	Polished	Polished
Area A	Floor 7	83	Wall		Partly ox.	7	Red slipped	Black burnished
Area A	Floor 7	-	Wall		Partly ox.	4	Eroded	Black burnished
Area A 100/105	Floor 7	84	Wall		Reduced	9	Red slipped	Black burnished
Area A 100/105	Floor 7	85	Wall		Reduced	7	Polished	Black burnished
Area A 100/105	Floor 7	86	Wall		Reduced	8	Red slipped	Polished
Area A 100/105	Floor 7	86	Wall		Oxyd.	5	Black burnished	Polished
Area A 100/105	Floor 7	87	Wall		Reduced	6	Polished	Polished
Area A 101/104	Floor 7	67	Wall		Reduced	8	Black burnished	Black burnished
Area A 101/105	Floor 7	68	Infl. point		Reduced	7	Polished	Polished
Area A 101/105	Floor 7	69	Wall		Reduced	6	Polished	Polished
Area A 101/105	Floor 7	88	Wall		Reduced	7	Red slipped	Polished
Area B 100/107	Floor 7	72	Wall		Reduced	6	Polished	Polished
Area B 100/107	Floor 7	73	Wall		Reduced	5	Polished	Polished
Area B 101/107	Floor 7	-	Wall		Reduced	7	Polished	Polished
Area B 101/107	Floor 7	71	Wall		Reduced	6	Polished	Black burnished
Area B 101/107	Floor 7	72	Wall		Reduced	5	Polished	Polished
Area B 101/108	Floor 7	22	Wall		Reduced	6	Eroded	Eroded
Area A	Floor 6	-	Wall		Reduced	8	Black burnished	Polished
Area A	Floor 6	-	Wall		Reduced	4	Polished	Polished
Area A	Floor 6	-	Wall		Reduced	4	Black burnished	Black burnished
Area A 101/104	Floor 6	91	Wall		Partly ox.	7	Eroded	Eroded
Area A 101/104	Floor 6	91	Infl. point		Partly ox.	5	Eroded	Black burnished
Area B 100/108	Floor 6	47	Wall		Oxyd.	10	Eroded	Eroded
Area B 100/108	Floor 6	48	Wall		Partly ox.	5	Black burnished	Black burnished
Area B 101/108	Floor 6	25	Wall		Partly ox.	10	Red slipped	Polished
Area B 101/108	Floor 6	-	Wall		Reduced	5	Eroded	Eroded
Area A	Floor 5	100	Wall		Reduced	7	Polished	Polished
Area A	Floor 5	101	Wall		Partly ox.	8	Eroded	Eroded
Area A	Floor 5	104	Wall		Partly ox.	8	Red slipped	Eroded
Area A	Floor 5	105	Wall		Reduced	7	Eroded	Eroded
Area A	Floor 5	108	Wall		Oxyd.	9	Red slipped	Polished
Area A	Floor 5	109	Wall		Partly ox.	7	Red slipped	Black burnished
Area A	Floor 5	110	Wall		Oxyd.	10	Eroded	Eroded
Area A	Floor 5	111	Wall		Reduced	7	Red slipped	Black burnished
Area A	Floor 5	113	Wall		Partly ox.	9	Eroded	Red slipped
Area A	Floor 5	115	Wall		Partly ox.	10	Red slipped	Black burnished
Area A	Floor 5	116	Wall		Reduced	12	Polished	Polished
Area A	Floor 5	116	Rim	20	Reduced	9	Polished	Polished
Area A	Floor 5	117	Wall		Reduced	9	Black burnished	Black burnished
Area A	Floor 5	117	Wall		Reduced	9	Black burnished	Black burnished
Area A	Floor 5	118	Wall		Oxyd.	10	Red slipped	Black burnished
Area A	Floor 5	119	Wall		Partly ox.	11	Polished	Eroded

Area A	Floor 5	120	Wall		Reduced	9	Black burnished	Red slipped
Area A	Floor 5	121	Wall		Oxyd.	14	Red slipped	Black burnished
Area A	Floor 5	122	Wall		Oxyd.	9	Red slipped	Polished
Area A	Floor 5	123	Wall		Partly ox.	8	Black burnished	Black burnished
Area A	Floor 5	124	Wall		Oxyd.	10	Eroded	Black burnished
Area A	Floor 5	125	Wall		Oxyd.	9	Red slipped	Black burnished
Area A	Floor 5	126	Wall		Partly ox.	7	Red slipped	Black burnished
Area A	Floor 5	127	Wall		Reduced	8	Red slipped	Black burnished
Area A	Floor 5	128	Wall		Reduced	7	Red slipped	Black burnished
Area A	Floor 5	128	Wall		Partly ox.	11	Red slipped	Black burnished
Area A	Floor 5	129	Wall		Partly ox.	8	Red slipped	Black burnished
Area A	Floor 5	130	Rim	14	Reduced	5	Black burnished	Polished
Area A	Floor 5	131	Wall		Reduced	8	Polished	Polished
Area A	Floor 5	132	Wall		Reduced	9	Polished	Black burnished
Area A	Floor 5	132	Wall		Reduced	5	Black burnished	Black burnished
Area A	Floor 5	133	Wall		Reduced	7	Polished	Polished
Area A	Floor 5	135	Wall		Partly ox.	5	Eroded	Polished
Area A	Floor 5	135	Wall		Partly ox.	12	Red slipped	Polished
Area A	Floor 5	137	Wall		Partly ox.	10	Red slipped	Black burnished
Area A	Floor 5	139	Rim	30	Reduced	8	Polished	Polished
Area A	Floor 5	141	Infl. point		Reduced	5	Polished	Polished
Area A	Floor 5	142	Rim	14	Reduced	6	Black burnished	Black burnished
Area A	Floor 5	143	Wall		Reduced	9	Red slipped	Black burnished
Area A	Floor 5	144	Wall		Reduced	9	Polished	Eroded
Area A	Floor 5	145	Rim	24	Reduced	10	Polished	Polished
Area A	Floor 5	146	Wall		Reduced	10	Red slipped	Black burnished
Area A	Floor 5	147	Wall		Reduced	10	Eroded	Polished
Area A	Floor 5	148	Wall		Reduced	8	Eroded	Eroded
Area A	Floor 5	149	Wall		Partly ox.	4	Punct.	Black burnished
Area A	Floor 5	150	Wall		Reduced	11	Red slipped	Black burnished
Area A	Floor 5	151	Infl. point		Partly ox.	5	Punctate	Black burnished
Area A	Floor 5	152	Rim	10	Reduced	5	Black burnished	Black burnished
Area A	Floor 5	153	Wall		Reduced	8	Eroded	Polished
Area A	Floor 5	154	Wall		Oxyd.	12	Eroded	Black burnished
Area A	Floor 5	154	Rim	14	Reduced	5	Polished	Polished
Area A	Floor 5	154	Rim	14	Reduced	5	Eroded	Eroded
Area A	Floor 5	155	Wall		Reduced	7	Eroded	Eroded
Area A	Floor 5	156	Wall		Partly ox.	13	Eroded	Eroded
Area A	Floor 5	156	Wall		Partly ox.	8	Eroded	Eroded
Area A	Floor 5	94	Wall		Partly ox.	12	Red slipped	Polished
Area A	Floor 5	95	Rim	24	Reduced	9	Black burnished	Polished
Area A	Floor 5	96	Wall		Reduced	13	Red slipped	Eroded
Area A	Floor 5	99	Wall		Partly ox.	5	Eroded	Eroded
Area A	Floor 5	99	Wall		Reduced	8	Red slipped	Black burnished
Area A	Floor 5	99	Wall		Reduced	9	Black burnished	Polished
Area A	Floor 5	99	Wall		Oxyd.	9	Eroded	Eroded
Area A	Floor 5	99	Wall		Partly ox.	10	Red slipped	Polished
Area A	Floor 5	99	Wall		Reduced	5	Eroded	Eroded
Area A	Floor 5	99	Wall		Reduced	8	Black burnished	Black burnished
Area A	Floor 5	99	Rim	16	Reduced	8	Polished	Polished

Area A	Floor 5	99	Rim	16	Reduced	9	Black burnished	Polished
Area A	Floor 5	-	Wall		Reduced	5	Polished	Polished
Area A	Floor 5	-	Wall		Reduced	4	Polished	Polished
Area A	Floor 5	-	Rim	8	Reduced	5	Polished	Polished
Area A	Floor 5	84	Wall		Reduced	8	Polished	Polished
Area A	Floor 5	79	Wall		Reduced	5	Red slipped	Black burnished
Area B 100/107	Floor 5	88	Infl. point		Reduced	10	Black burnished	Polished
Area B 100/107	Floor 5	91	Wall		Reduced	6	Black burnished	Black burnished
Area B 100/107	Floor 5	-	Wall		Partly ox.	7	Eroded	Black burnished
Area B 100/107	Floor 5	-	Wall		Reduced	5	Eroded	Eroded
Area B 100/107	Floor 5	80	Wall		Partly ox.	6	Red slipped	Eroded
Area B 100/107	Floor 5	-	Wall		Reduced	9	Red slipped	Black burnished
Area B 100/107	Floor 5	100	Wall		Reduced	7	Black burnished	Black burnished
Area B 100/108	Floor 5	53	Wall		Reduced	9	Black burnished	Eroded
Area B 100/108	Floor 5	54	Wall		Partly ox.	8	Red slipped	Black burnished
Area B 100/108	Floor 5	55	Wall		Reduced	10	Red slipped	Black burnished
Area B 100/108	Floor 5	56	Wall		Partly ox.	15	Red slipped	Black burnished
Area B 100/108	Floor 5	57	Wall		Oxyd.	8	Red slipped	Polished
Area B 100/108	Floor 5	53	Wall		Reduced	9	Red slipped	Black burnished
Area B 100/108	Floor 5	54	Wall		Reduced	9	Black burnished	Polished
Area B 100/108	Floor 5	56	Wall		Reduced	10	Polished	Polished
Area B 100/108	Floor 5	57	Wall		Reduced	10	Black burnished	Polished
Area B 100/108	Floor 5	57	Rim		Reduced	10	Eroded	Eroded
Area B 100/108	Floor 5	51	Wall		Reduced	7	Polished	Black burnished
Area B 101/107	Floor 5	76	Wall		Reduced	5	Black burnished	Black burnished
Area B 101/107	Floor 5	77	Wall		Reduced	10	Polished	Polished
Area B 101/107	Floor 5	78	Infl. point		Reduced	5	Polished	Black burnished
Area B 101/107	Floor 5	79	Wall		Reduced	6	Polished	Black burnished
Area B 101/108	Floor 5	34	Wall		Reduced	6	Polished	Polished
Area B 101/108	Floor 5	35	Wall		Reduced	8	Black burnished	Black burnished
Area B 101/108	Floor 5	36	Infl. point		Reduced	6	Black burnished	Polished
Area B 101/108	Floor 5	38	Wall		Reduced	6	Black burnished	Black burnished
Area B 101/108	Floor 5	39	Wall		Reduced	9	Eroded	Eroded
Area B 101/108	Floor 5	40	Wall		Reduced	9	Polished	Polished
Area B 101/108	Floor 5	41	Base		Reduced	16	Black burnished	Black burnished
Area B 101/108	Floor 5	43	Wall		Reduced	9	Black burnished	Black burnished
Area B 101/108	Floor 5	45	Wall		Reduced	9	Polished	Black burnished
Area B 101/108	Floor 5	46	Wall		Reduced	7	Black burnished	Black burnished
Area B 101/108	Floor 5	47	Wall		Reduced	8	Black burnished	Black burnished
Area B 101/108	Floor 5	48	Wall		Reduced	9	Black burnished	Black burnished
Area B 101/108	Floor 5	50	Wall		Reduced	6	Polished	Red slipped
Area B 101/108	Floor 5	51	Wall		Reduced	7	Polished	Black burnished
Area B 101/108	Floor 5	52	Wall		Reduced	8	Polished	Black burnished
Area B 101/108	Floor 5	53	Wall		Reduced	8	Black burnished	Polished
Area A	Floor 4	161	Wall		Reduced	5	Black burnished	Black burnished
Area A	Floor 4	163	Wall		Reduced	5	Black burnished	Polished
Area A	Floor 4	164	Wall		Reduced	8	Polished	Black burnished
Area A	Floor 4	157	Rim	16	Reduced	4	Black burnished	Black burnished
Area A	Floor 4	158	Wall		Partly ox.	9	Red slipped	Polished
Area A	Floor 4	159	Wall		Reduced	9	Black burnished	Black burnished

Area A	Floor 4	160	Wall		Reduced	11	Red slipped	Polished
Area A	Floor 4	168	Wall		Partly ox.	5	Black burnished	Black burnished
Area A	Floor 4	169	Wall		Reduced	10	Eroded	Eroded
Area A	Floor 4	171	Wall		Reduced	5	Polished	Polished
Area A	Floor 4	172	Wall		Reduced	5	Polished	Polished
Area A	Floor 4	173	Rim	10	Reduced	5	Polished	Polished
Area A	Floor 4	174	Wall		Reduced	6	Eroded	Eroded
Area A	Floor 4	180	Base		Partly ox.	6	Red slipped	Black burnished
Area A	Floor 4	181	Rim	12	Reduced	5	Black burnished	Black burnished
Area A	Floor 4	186	Infl. point		Reduced	6	Black burnished	Black burnished
Area A	Floor 4	187	Wall		Reduced	10	Black burnished	Eroded
Area A	Floor 4	191	Wall		Reduced	6	Polished	Polished
Area A	Floor 4	192	Wall		Partly ox.	5	Red slipped	Black burnished
Area A	Floor 4	193	Rim	11	Reduced	4	Black burnished	Polished
Area A	Floor 4	194	Wall		Partly ox.	7	Polished	Polished
Area A	Floor 4	195	Wall		Oxyd.	13	Eroded	Eroded
Area A	Floor 4	196	Wall		Reduced	6	Polished	Black burnished
Area A	Floor 4	197	Wall		Reduced	9	Black burnished	Black burnished
Area A	Floor 4	198	Wall		Partly ox.	8	Red slipped	Black burnished
Area A	Floor 4	-	Wall		Reduced	5	Black burnished	Black burnished
Area A	Floor 4	190	Wall		Reduced	8	Red slipped	Polished
Area A	Floor 4	177	Wall		Partly ox.	13	Red slipped	Eroded
Area A	Floor 4	178	Wall		Reduced	9	Red slipped	Polished
Area A	Floor 4	189	Wall		Reduced	7	Black burnished	Black burnished
Area A	Floor 4	206	Wall		Reduced	8	Polished	Black burnished
Area A	Floor 4	211	Wall		Reduced	6	Polished	Black burnished
Area A	Floor 4	212	Wall		Reduced	8	Black burnished	Black burnished
Area A	Floor 4	213	Wall		Reduced	5	Eroded	Eroded
Area A	Floor 4	207	Wall		Reduced	8	Polished	Black burnished
Area A	Floor 4	214	Wall		Reduced	7	Polished	Polished
Area A	Floor 4	216	Wall		Reduced	5	Polished	Black burnished
Area A	Floor 4	218	Rim	20	Oxyd.	7	Black burnished	Black burnished
Area A	Floor 4	219	Wall		Reduced	6	Polished	Black burnished
Area A	Floor 4	209	Wall		Reduced	6	Black burnished	Polished
Area A	Floor 4	209	Wall		Oxyd.	6	Red slipped	Black burnished
Area A	Floor 4	209	Infl. point		Reduced	6	Black burnished	Black burnished
Area A	Floor 4	210	Wall		Reduced	6	Eroded	Black burnished
Area A	Floor 4	179	Wall		Reduced	5	Black burnished	Black burnished
Area A	Floor 4	179	Wall		Reduced	6	Black burnished	Black burnished
Area A	Floor 4	208	Wall		Reduced	9	Black burnished	Black burnished
Area B	Floor 4	92	Infl. point		Reduced	8	Polished	Black burnished
Area B	Floor 4	-	Wall		Reduced	5	Black burnished	Black burnished
Area B 100/107	Floor 4	98	Wall		Reduced	6	Eroded	Eroded
Area B 100/107	Floor 4	99	Wall		Reduced	6	Eroded	Eroded
Area B 100/107	Floor 4	97	Wall		Reduced	5	Eroded	Eroded
Area B 100/107	Floor 4	101	Wall		Reduced	9	Polished	Polished
Area B 100/107	Floor 4	102	Wall		Reduced	6	Black burnished	Black burnished
Area B 100/107	Floor 4	103	Wall		Partly ox.	16	Red slipped	Eroded
Area B 100/107	Floor 4	108	Wall		Reduced	4	Black burnished	Black burnished
Area B 100/107	Floor 4	-	Wall		Reduced	5	Polished	Black burnished

Area B 100/107	Floor 4	106	Base		Reduced	4	Black burnished	Black burnished
Area B 100/107	Floor 4	111	Infl. point		Reduced	4	Black burnished	Black burnished
Area B 100/107	Floor 4	87	Base		Reduced	4	Black burnished	Black burnished
Area B 100/108	Floor 4	58	Wall		Reduced	5	Eroded	Eroded
Area B 100/108	Floor 4	59	Wall		Reduced	8	Polished	Polished
Area B 100/108	Floor 4	60	Wall		Reduced	7	Red slipped	Black burnished
Area B 100/108	Floor 4	61	Wall		Reduced	6	Polished	Polished
Area B 100/108	Floor 4	62	Wall		Reduced	5	Black burnished	Black burnished
Area B 100/108	Floor 4	63	Wall		Reduced	6	Black burnished	Eroded
Area B 100/108	Floor 4	64	Wall		Reduced	7	Black burnished	Black burnished
Area B 100/108	Floor 4	65	Wall		Reduced	8	Black burnished	Black burnished
Area B 100/108	Floor 4	-	Wall		Reduced	4	Black burnished	Black burnished
Area B 100/108	Floor 4	66	Wall		Reduced	8	Black burnished	Black burnished
Area B 100/108	Floor 4	67	Wall		Reduced	8	Eroded	Polished
Area B 101/107	Floor 4	82	Wall		Partly ox.	8	Red slipped	Black burnished
Area B 101/107	Floor 4	82	Wall		Reduced	10	Polished	Polished
Area B 101/107	Floor 4	83	Wall		Reduced	7	Black burnished	Black burnished
Area B 101/107	Floor 4	84	Wall		Reduced	6	Nail impressed op. dir	Black burnished
Area B 101/107	Floor 4	89	Wall		Reduced	7	Pinched	Black burnished
Area B 101/107	Floor 4	97	Wall		Reduced	6	Black burnished	Black burnished
Area B 101/107	Floor 4	100	Wall		Reduced	7	Incised	Eroded
Area B 101/108	Floor 4	55	Wall		Reduced	14	Red slipped	Black burnished
Area B 101/108	Floor 4	57	Wall		Reduced	5	Black burnished	Polished
Area B 101/108	Floor 4	58	Wall		Reduced	6	Red slipped	Black burnished
Area A	Floor 3	200	Wall		Oxyd.	8	Eroded	Eroded
Area A	Floor 3	201	Wall		Oxyd.	6	Polished	Eroded
Area A	Floor 3	202	Wall		Reduced	4	Black burnished	Black burnished
Area A	Floor 3	204	Wall		Reduced	5	Black burnished	Polished
Area A	Floor 3	220	Infl. point		Reduced	4	Incised	Polished
Area A	Floor 3	221	Rim	5	Reduced	4	Nail impressed	Eroded
Area A	Floor 3	222	Base		Reduced	8	Black burnished	Black burnished
Area A	Floor 3	223	Rim	12	Reduced	4	Black burnished	Polished
Area A	Floor 3	224	Wall		Reduced	8	Red slipped	Black burnished
Area A	Floor 3	224	Rim		Reduced	6	Black burnished	Black burnished
Area A	Floor 3	226	Rim	19	Reduced	6	Polished	Polished
Area A	Floor 3	228	Rim		Reduced	7	Eroded	Eroded
Area A	Floor 3	230	Infl. point		Reduced	7	Black burnished	Black burnished
Area A	Floor 3	231	Wall		Reduced	13	Polished	Black burnished
Area A	Floor 3	233	Wall		Oxyd.	12	Eroded	Eroded
Area A	Floor 3	233	Rim	10	Reduced	5	Eroded	Polished
Area A	Floor 3	235	Rim	15	Reduced	5	Black burnished	Eroded
Area A	Floor 3	236	Wall		Reduced	7	Polished	Black burnished
Area A	Floor 3	237	Wall		Reduced	5	Polished	Black burnished
Area A	Floor 3	238	Wall		Partly ox.	10	Red slipped	Black burnished
Area A	Floor 3	239	Wall		Reduced	13	Polished	Black burnished
Area A	Floor 3	239	Wall		Reduced	9	Red slipped	Polished
Area A	Floor 3	241	Rim	28	Reduced	9	Black burnished	Eroded
Area A	Floor 3	245	Wall		Reduced	7	Polished	Polished
Area A	Floor 3	251	Wall		Partly ox.	8	Red slipped	Black burnished
Area A	Floor 3	253	Wall		Reduced	15	Black burnished	Polished

Area A	Floor 3	254	Wall		Reduced	8	Black burnished	Polished
Area A	Floor 3	255	Infl. point		Reduced	6	Black burnished	Black burnished
Area A	Floor 3	255	Rim	28	Reduced	9	Black burnished	Polished
Area A	Floor 3	256	Wall		Reduced	5	Incised	Black burnished
Area A	Floor 3	257	Wall		Partly ox.	12	Red slipped	Polished
Area A	Floor 3	-	Wall		Reduced	8	Polished	Polished
Area A	Floor 3	-	Wall		Partly ox.	13	Eroded	Eroded
Area A	Floor 3	-	Wall		Partly ox.	7	Polished	Black burnished
Area A	Floor 3	-	Wall		Reduced	12	Black burnished	Polished
Area A	Floor 3	-	Rim	26	Oxyd.	9	Red slipped	Red slipped
Area A	Floor 3	-	Wall		Reduced	3	Punct.	Polished
Area A	Floor 3	199	Rim	20	Reduced	9	Polished	Polished
Area A	Floor 3	-	Wall		Reduced	7	Black burnished	Black burnished
Area A	Floor 3	-	Wall		Reduced	7	Polished	Black burnished
Area A	Floor 3	226	Rim	20	Partly ox.	5	Black burnished	Polished
Area A	Floor 3	240	Wall		Reduced	5	Polished	Polished
Area B	Floor 3	203	Wall		Oxyd.	9	Red slipped	Eroded
Area B	Floor 3	207	Wall		Reduced	7	Black burnished	Eroded
Area B	Floor 3	208	Wall		Partly ox.	5	Incised	Eroded
Area B	Floor 3	209	Wall		Partly ox.	5	Polished	Eroded
Area B	Floor 3	210	Wall		Reduced	10	Red slipped	Eroded
Area B	Floor 3	212	Wall		Reduced	9	Black burnished	Eroded
Area B	Floor 3	214	Wall		Partly ox.	9	Red slipped	Eroded
Area B	Floor 3	218	Wall		Reduced	9	Polished	Eroded
Area B	Floor 3	219	Wall		Partly ox.	10	Red slipped	Eroded
Area B	Floor 3	223	Wall		Reduced	6	Polished	Eroded
Area B	Floor 3	224	Rim	24	Reduced	5	Incised	Eroded
Area B	Floor 3	225	Wall		Reduced	5	Stamped	Eroded
Area B	Floor 3	226	Wall		Reduced	11	Red slipped	Eroded
Area B	Floor 3	227	Wall		Reduced	6	Red slipped	Eroded
Area B	Floor 3	235	Wall		Partly ox.	8	Red slipped	Eroded
Area B	Floor 3	236	Wall		Reduced	6	Polished	Eroded
Area B	Floor 3	239	Wall		Reduced	9	Eroded	Eroded
Area B	Floor 3	240	Wall		Reduced	5	Black burnished	Eroded
Area B	Floor 3	241	Wall		Oxyd.	8	Red slipped	Eroded
Area B	Floor 3	242	Rim		Reduced	7	Black burnished	Eroded
Area B	Floor 3	242	Infl. point		Reduced	4	Black burnished	Eroded
Area B	Floor 3	243	Wall		Reduced	5	Stamped	Eroded
Area B	Floor 3	244	Wall		Reduced	7	Polished	Eroded
Area B	Floor 3	245	Wall		Reduced	9	Red slipped	Eroded
Area A	Floor 2	258	Wall		Reduced	5	Polished	Black burnished
Area A	Floor 2	259	Rim	30	Reduced	11	Black burnished	Black burnished
Area A	Floor 2	259	Rim		Reduced	5	Polished	Polished
Area B	Floor 2	249	Wall		Reduced	11	Polished	Black burnished
Area B	Floor 2	250	Wall		Reduced	8	Eroded	Eroded
Area B	Floor 2	254	Wall		Partly ox.	11	Eroded	Black burnished
Area B	Floor 2	269	Wall		Reduced	9	Polished	Black burnished
Area B	Floor 2	270	Wall		Reduced	4	Pinched	Black burnished
Area B	Floor 2	274	Wall		Partly ox.	9	Red slipped	Black burnished
Area B	Floor 2	279	Wall		Partly ox.	15	Red slipped	Black burnished

Area B	Floor 2	279	Wall	Partly ox.	15	Red slipped	Black burnished
Area B	Floor 2	279	Wall	Partly ox.	15	Red slipped	Black burnished
Area B	Floor 2	279	Wall	Partly ox.	15	Red slipped	Black burnished
Area B	Floor 2	279	Wall	Partly ox.	15	Red slipped	Black burnished
Area B	Floor 2	279	Wall	Partly ox.	15	Red slipped	Black burnished
Area B	Floor 2	279	Wall	Partly ox.	15	Red slipped	Black burnished
Area B	Floor 2	279	Wall	Partly ox.	15	Red slipped	Black burnished
Area B	Floor 2	279	Wall	Partly ox.	15	Red slipped	Black burnished
Area B	Floor 2	279	Wall	Partly ox.	15	Red slipped	Black burnished
Area B	Floor 2	279	Wall	Partly ox.	15	Red slipped	Black burnished
Area B	Floor 2	279	Wall	Partly ox.	15	Red slipped	Black burnished
Area B	Floor 2	279	Wall	Partly ox.	15	Red slipped	Black burnished
Area B	Floor 2	280	Wall	Reduced	12	Black burnished	Black burnished
Area B	Floor 2	281	Wall	Reduced	11	Polished	Polished
Area B 101/107	Floor 2	246	Wall	Partly ox.	11	Red slipped	Black burnished
Area B 101/107	Floor 2	247	Wall	Reduced	10	Black burnished	Black burnished
Area B 101/107	Floor 2	248	Rim	Reduced	6	Black burnished	Black burnished
Area A	Floor 1	260	Wall	Reduced	8	Stamped	Black burnished
Area A	Floor 1	263	Wall	Partly ox.	5	Polished	Black burnished
Area A	Floor 1	264	Wall	Partly ox.	7	Polished	Black burnished
Area A	Floor 1	-	Wall	Reduced	7	Punct.	Black burnished
Area B	Floor 1	300	Wall	Reduced	6	Eroded	Eroded
Area B	Floor 1	301	Wall	Partly ox.	8	Eroded	Eroded
Area B	Floor 1	302	Base	Reduced	5	Black burnished	Eroded
Area B	Floor 1	303	Infl. point	Reduced	8	Black burnished	Black burnished
Area B	Floor 1	304	Wall	Reduced	6	Black burnished	Black burnished
Area B	Floor 1	305	Wall	Reduced	10	Eroded	Black burnished
Area B	Floor 1	306	Wall	Reduced	7	Polished	Black burnished
Area B	Floor 1	311	Wall	Reduced	6	Black burnished	Black burnished
Area B	Floor 1	313	Wall	Reduced	7	Stamped	Black burnished
Area B	Floor 1	314	Wall	Reduced	5	Eroded	Black burnished
Area B	Floor 1	315	Wall	Reduced	7	Polished	Black burnished
Area B	Floor 1	316	Wall	Reduced	5	Eroded	Black burnished
