# Supplementary material for: Understanding the Chronology and Occupation Dynamics of Oversized Pit Houses in the Southern Brazilian Highlands
Source: PLoS One. 2016 Jul 6;11(7):e0158127. doi: 10.1371/journal.pone.0158127 (PMC4934860; doi:10.1371/journal.pone.0158127)
Supplement: S2 Table — (PDF) [file pone.0158127.s003.pdf]

	Ceramic types		
**Stratum**	**Plain**	**Red**	**Decorated**
Floor 12	27	2	4
Floor 11	16	4	0
Floor 10	1	0	0
Floor 9	5	0	0
Floor 8	14	5	1
Floor 7	13	4	0
Floor 6	4	1	0
Floor 5	56	31	2
Floor 4	53	14	3
Floor 3	37	15	8
Floor 2	9	15	1
Floor 1	8	0	3
