# Supplementary material for: Understanding the Chronology and Occupation Dynamics of Oversized Pit Houses in the Southern Brazilian Highlands
Source: PLoS One. 2016 Jul 6;11(7):e0158127. doi: 10.1371/journal.pone.0158127 (PMC4934860; doi:10.1371/journal.pone.0158127)
Supplement: S3 Table — (PDF) [file pone.0158127.s004.pdf]

Ceramic types	Burning atmosphere			Thickness (mm)			Use wear	
	Reduced	Incompletely oxidised	Oxidised	3-5	6-10	11-16	Carbonised residue	No residue
Plain	312	30	12	115	226	13	25	328
Red	39	56	14	7	73	29	44	65
Decorated	26	7	0	23	10	0	0	33
