# Supplementary material for: Understanding the Chronology and Occupation Dynamics of Oversized Pit Houses in the Southern Brazilian Highlands
Source: PLoS One. 2016 Jul 6;11(7):e0158127. doi: 10.1371/journal.pone.0158127 (PMC4934860; doi:10.1371/journal.pone.0158127)
Supplement: S4 Table — (PDF) [file pone.0158127.s005.pdf]

Unit	Find #	Stratum	Type	Raw material	Modif.	Dimensions (cm)
Area A 100/104	20	Floor 12	Flake	Quartz		1.6 x 1.6
Area A 101/104	16	Floor 12	Flake	Quartz	Retouch	3 x 1.4
Area A 101/104	18	Floor 12	Core	Basalt		12.3 x 13.4 x 10.2
Area B 100/107	38	Floor 12	Debris	Chert		
Area B 100/107	39	Floor 12	Flake	Quartz		1 x 0.9
Area B 100/107	55	Floor 12	Flake	Chert		4.4 x 2.7
Area B 100/107	-	Floor 12	Flake	Quartz		1.1 x 0.9
Area B 100/108	19	Floor 12	Flake	Chert	Use wear	2.2 x 2
Area B 100/108	21	Floor 12	Flake	Chert		2 x 2
Area B 101/107	28	Floor 12	Flake	Chert		2.6 x 1.5
Area B 101/107	36	Floor 12	Uniface	Basalt	Retouch	8 x 7.9 x 4.2
Area B 101/107	39	Floor 12	Debris	Basalt		
Area B 101/107	46	Floor 12	Flake	Basalt		9.8 x 7
Area A 100/104	30	Floor 11	Flake	Basalt		4.5 x 4
Area A 100/105	28	Floor 11	Flake	Quartz		1.4 x 0.7
Area A 100/105	30	Floor 11	Flake	Chert		2.6 x 2.6
Area A 101/104	29	Floor 11	Flake	Basalt	Retouch	7.3 x 5.1
Area B 101/107	51	Floor 11	Flake	Quartz	Use wear	2 x 1.2
Area B 101/107	-	Floor 11	Debris	Quartz		
Area A 101/105	-	Floor 10	Debris	Quartz		
Area A 100/104	42	Floor 9	Flake	Chert		2.3 x 1.9
Area A 100/105	64	Floor 8	Flake	Basalt	Use wear	8.1 x 7.7
Area B 100/107	71	Floor 8	Flake	Basalt		3 x 3.2
Area A 101/105	80	Floor 8	Flake	Quartz		3.2 x 1.6
Area A	55	Floor 8	Flake	Quartz	Use wear	3 x 1.9
Area A	60	Floor 8	Flake	Basalt	Retouch	6.7 x 4.2
Area A	-	Floor 8	Flake	Chert	Retouch	3.5 x 2.9
Area A	-	Floor 8	Flake	Chert		2 x 1
Area B 101/107	70	Floor 7	Flake	Quartz	Use wear	3.1 x 1.3
Area A	66	Floor 7	Flake	Quartz		1.4 x 1.3
Area A	74	Floor 7	Flake	Quartz	Retouch	2.1 x 1.2
Area A	76	Floor 7	Debris	Chert		
Area A	82	Floor 7	Flake	Basalt	Retouch	8.1 x 6.6
Area A	-	Floor 7	Flake	Quartz		1.9 x 1.3
Area A	-	Floor 7	Flake	Chert		2.2 x 1.5
Area A	-	Floor 7	Flake	Basalt		4.6 x 4.8
Area B 100/108	50	Floor 6	Core	Basalt		12.2 x 9.2 x 7.1
Area A	103	Floor 5	Flake	Basalt		7.3 x 5.2
Area A	114	Floor 5	Flake	Basalt		4.8 x 4.5
Area A	138	Floor 5	Flake	Basalt		7.8 x 5.5
Area A	140	Floor 5	Debris	Chert		
Area B 100/107	79	Floor 5	Flake	Quartz		1.8 x 1
Area B 101/108	30	Floor 5	Flake	Quartz		1.7 x 2.3
Area A	167	Floor 5	Flake	Basalt		8.6 x 8
Area A	-	Floor 5	Flake	Basalt		2.3 x 4.3
Area A	93	Floor 5	Core	Quartz		1 x 1.5 x 2.5
Area A 100/104	92	Floor 5	Debris	Quartz		
Area B 100/107	96	Floor 4	Flake	Basalt		6.2 x 4.9
Area B 100/107	-	Floor 4	Flake	Basalt		6.4 x 3.4

Area B 100/107	107	Floor 4	Core	Basalt		13.2 x 8 x 8.4
Area B 100/107	110	Floor 4	Debris	Chert		
Area B	88	Floor 4	Core	Basalt		15.7 x 15.7 x 9.5
Area B 101/108	56	Floor 4	Flake	Basalt		4.1 x 4.5
Area A	88	Floor 4	Flake	Basalt	Retouch	6.9 x 6
Area A	8	Floor 4	Debris	Basalt		
Area A	17	Floor 4	Flake	Basalt	Use wear	5 x 3
Area A	17	Floor 4	Flake	Basalt		1.5 x 3.2
Area A	17	Floor 4	Flake	Chert		1.6 x 1.9
Area B 100/107	94	Floor 4	Core	Chert		4.4 x 3.3 x 2.2
Area B 100/107	-	Floor 4	Flake	Chert	Use wear	3.5 x 1.3
Area B	202	Floor 3	Flake	Chert	Use wear	4.5 x 3.5
Area B	216	Floor 3	Flake	Basalt		8.7 x 5.8
Area B	217	Floor 3	Flake	Basalt		8.9 x 7.1
Area B	238	Floor 3	Biface	Basalt		13.2 x 7.5 x 4.4
Area A	232	Floor 3	Flake	Basalt		4.3 x 3.3
Area A	226	Floor 3	Flake	Basalt		2.9 x 5.3
Area A	243	Floor 3	Column	Basalt	Retouch	18 x 5.7 x 4.5
Area A	244	Floor 3	Column	Basalt	Retouch	6.9 x 3 x 2
Area B	271	Floor 2	Flake	Basalt		7.6 x 10.7
Area A	261	Floor 1	Basalt	Flake		3.4 x 2.5
Area A	261	Floor 1	Basalt	Debris		
Area A	-	Floor 1	Quartz	Flake		1.5 x 1.6
