# Supplementary material for: Understanding the Chronology and Occupation Dynamics of Oversized Pit Houses in the Southern Brazilian Highlands
Source: PLoS One. 2016 Jul 6;11(7):e0158127. doi: 10.1371/journal.pone.0158127 (PMC4934860; doi:10.1371/journal.pone.0158127)
Supplement: S5 Table — (PDF) [file pone.0158127.s006.pdf]

			Technological types	
**Stratum**	**Flakes**	**Cores**	**Bifacial / unifacial tools**	**Utilised / retouched flakes**
Floor 12	7	1	1	2
Floor 11	3	0	0	2
Floor 10	0	0	0	0
Floor 9	1	0	0	0
Floor 8	3	0	0	4
Floor 7	4	0	0	3
Floor 6	0	1	0	0
Floor 5	7	1	0	0
Floor 4	5	3	0	3
Floor 3	4	0	3	1
Floor 2	1	0	0	0
Floor 1	2	0	0	0
