# Supplementary material for: Understanding the Chronology and Occupation Dynamics of Oversized Pit Houses in the Southern Brazilian Highlands
Source: PLoS One. 2016 Jul 6;11(7):e0158127. doi: 10.1371/journal.pone.0158127 (PMC4934860; doi:10.1371/journal.pone.0158127)
Supplement: S6 Table — (PDF) [file pone.0158127.s007.pdf]

	Raw material		
Stratum	Basalt	Quartz	Chert
Floor 12	3	4	5
Floor 11	2	3	1
Floor 10	0	1	0
Floor 9	0	0	1
Floor 8	3	2	2
Floor 7	2	4	2
Floor 6	1	0	0
Floor 5	5	4	1
Floor 4	9	0	4
Floor 3	7	0	1
Floor 2	1	0	0
Floor 1	2	1	0
